# The epidemiology of adolescents living with perinatally acquired HIV: A cross-region global cohort analysis

**DOI:** 10.1371/journal.pmed.1002514

**Published:** 2018-03-01

**Authors:** The Collaborative Initiative for Paediatric HIV Education and Research (CIPHER) Global Cohort Collaboration, Amy L. Slogrove, Michael Schomaker, Mary-Ann Davies, Paige Williams, Suna Balkan, Jihane Ben-Farhat, Nancy Calles, Kulkanya Chokephaibulkit, Charlotte Duff, Tanoh François Eboua, Adeodata Kekitiinwa-Rukyalekere, Nicola Maxwell, Jorge Pinto, George Seage, Chloe A. Teasdale, Sebastian Wanless, Josiane Warszawski, Kara Wools-Kaloustian, Marcel Yotebieng, Venessa Timmerman, Intira J. Collins, Ruth Goodall, Colette Smith, Kunjal Patel, Mary Paul, Diana Gibb, Rachel Vreeman, Elaine J. Abrams, Rohan Hazra, Russell Van Dyke, Linda-Gail Bekker, Lynne Mofenson, Marissa Vicari, Shaffiq Essajee, Martina Penazzato, Gabriel Anabwani, Edith Q. Mohapi, Peter N. Kazembe, Makhosazana Hlatshwayo, Mwita Lumumba, Tessa Goetghebuer, Claire Thorne, Luisa Galli, Annemarie van Rossum, Carlo Giaquinto, Magdalena Marczynska, Laura Marques, Filipa Prata, Luminita Ene, Liubov Okhonskaia, Pablo Rojo, Claudia Fortuny, Lars Naver, Christoph Rudin, Sophie Le Coeur, Alla Volokha, Vanessa Rouzier, Regina Succi, Annette Sohn, Azar Kariminia, Andrew Edmonds, Patricia Lelo, Samuel Ayaya, Patricia Ongwen, Laura F. Jefferys, Sam Phiri, Mwangelwa Mubiana-Mbewe, Shobna Sawry, Lorna Renner, Mariam Sylla, Mark J. Abzug, Myron Levin, James Oleske, Miriam Chernoff, Shirley Traite, Murli Purswani, Ellen G. Chadwick, Ali Judd, Valériane Leroy

**Affiliations:** 1 Center for Infectious Diseases Epidemiology and Research, University of Cape Town, Cape Town, South Africa; 2 Harvard T. H. Chan School of Public Health, Boston, Massachusetts, United States of America; 3 Epicentre, Médecins Sans Frontières, Paris, France; 4 Baylor International Pediatric AIDS Initiative, Texas Children’s Hospital-USA, Houston, Texas, United States of America; 5 Faculty of Medicine, Siriraj Hospital, Mahidol University, Bangkok, Thailand; 6 MRC Clinical Trials Unit at University College London, London, United Kingdom; 7 Yopougon University Hospital, University Félix Houphouët-Boigny, Abidjan, Côte d'Ivoire; 8 Baylor International Pediatric AIDS Initiative, Kampala, Uganda; 9 School of Medicine, Federal University of Minas Gerais, Belo Horizonte, Brazil; 10 ICAP at Columbia University Mailman School of Public Health, New York, New York, United States of America; 11 Inserm (French Institute of Health and Medical Research), CESP UMR Villejuif, France; 12 Indiana University School of Medicine, Indianapolis, Indiana, United States of America; 13 College of Public Health, Ohio State University, Columbus, Ohio, United States of America; 14 National Institute of Child Health and Human Development (NICHD), US National Institutes of Health, Rockville, Maryland, United States of America; 15 Tulane University, New Orleans, Louisiana, United States of America; 16 Desmond Tutu HIV Centre, University of Cape Town, Cape Town, South Africa; 17 Elizabeth Glaser Pediatric AIDS Foundation, Washington, DC, United States of America; 18 International AIDS Society, Geneva, Switzerland; 19 UNICEF, New York, New York, United States of America; 20 World Health Organization, Geneva, Switzerland; 21 Baylor International Pediatric AIDS Initiative, Gaborone, Botswana; 22 Baylor International Pediatric AIDS Initiative, Maseru, Lesotho; 23 Baylor International Pediatric AIDS Initiative, Lilongwe, Malawi; 24 Baylor International Pediatric AIDS Initiative, Mbabane, Swaziland; 25 Baylor International Pediatric AIDS Initiative, Mbeya, Tanzania; 26 Hospital St Pierre Cohort, Bruxelles, Belgium; 27 Institute of Child Health, University College London, London, United Kingdom; 28 Department of Health Sciences, University of Florence, Florence, Italy; 29 Erasmus MC University Medical Center Rotterdam-Sophia Children’s Hospital, Rotterdam, the Netherlands; 30 PENTA Foundation, Padova, Italy; 31 Medical University of Warsaw, Hospital of Infectious Diseases in Warsaw, Warsaw, Poland; 32 Centro Hospitalar do Porto, Porto, Portugal; 33 Hospital de Santa Maria/CHLN, Lisbon, Portugal; 34 Victor Babes Hospital, Bucharest, Romania; 35 Republican Hospital of Infectious Diseases, St Petersburg, Russian Federation; 36 Hospital Doce de Octubre, Madrid, Spain; 37 Hospital Sant Joan de Déu, Universitat de Barcelona, Barcelona, Spain; 38 Karolinska University Hospital, Stockholm, Sweden; 39 University Children’s Hospital, Basel, Switzerland; 40 Institut de Recherche pour le Développement (IRD) 174/PHPT, Faculty of Associated Medical Sciences, Chiang Mai University, Chiang Mai, Thailand; 41 Institut National d'Etudes Démograhiques (Ined), F-75020 Paris, France; 42 Shupyk National Medical Academy of Postgraduate Education, Kiev, Ukraine; 43 GHESKIO Center, Port-au-Prince, Haiti; 44 Universidade Federal de São Paulo, São Paulo, Brazil; 45 TREAT Asia/amfAR, Bangkok, Thailand; 46 Kirby Institute, UNSW, Sydney, Australia; 47 Gillings School of Global Public Health, The University of North Carolina at Chapel Hill, Chapel Hill, North Carolina, United States of America; 48 Pediatric Hospital Kalembe Lembe, Lingwala, Kinshasa, Democratic Republic of Congo; 49 Academic Model Providing Access to Healthcare (AMPATH), Eldoret, Kenya; 50 Family AIDS Care and Education Services, Kenya Medical Research Institute, Kisumu, Kenya; 51 SolidarMed Lesotho, Mozambique and Zimbabwe, Lucerne, Switzerland; 52 Lighthouse Trust Clinic, Lilongwe, Malawi; 53 Center for Infectious Disease Research in Zambia, Lusaka, Zambia; 54 Wits Reproductive Health and HIV Institute, Faculty of Health Sciences, University of the Witwatersrand, Johannesburg, South Africa; 55 Harriet Shezi Children’s Clinic, Chris Hani Baragwanath Hospital, Johannesburg, South Africa; 56 University of Ghana School of Medicine and Dentistry, Accra, Ghana; 57 CHU Gabriel Touré, Bamako, Mali; 58 University of Colorado School of Medicine and Children’s Hospital Colorado, Aurora, Colorado, United States of America; 59 Rutgers New Jersey Medical School, Newark, New Jersey, United States of America; 60 Bronx-Lebanon Hospital Center (Icahn School of Medicine at Mount Sinai), Bronx, New York, United States of America; 61 Feinberg School of Medicine, Northwestern University, Evanston, Illinois, United States of America; 62 Inserm (French Institute of Health and Medical Research), UMR 1027 Université Toulouse 3, Toulouse, France; San Francisco General Hospital, UNITED STATES

## Abstract

**Background:**

Globally, the population of adolescents living with perinatally acquired HIV (APHs) continues to expand. In this study, we pooled data from observational pediatric HIV cohorts and cohort networks, allowing comparisons of adolescents with perinatally acquired HIV in “real-life” settings across multiple regions. We describe the geographic and temporal characteristics and mortality outcomes of APHs across multiple regions, including South America and the Caribbean, North America, Europe, sub-Saharan Africa, and South and Southeast Asia.

**Methods and findings:**

Through the Collaborative Initiative for Paediatric HIV Education and Research (CIPHER), individual retrospective longitudinal data from 12 cohort networks were pooled. All children infected with HIV who entered care before age 10 years, were not known to have horizontally acquired HIV, and were followed up beyond age 10 years were included in this analysis conducted from May 2016 to January 2017. Our primary analysis describes patient and treatment characteristics of APHs at key time points, including first HIV-associated clinic visit, antiretroviral therapy (ART) start, age 10 years, and last visit, and compares these characteristics by geographic region, country income group (CIG), and birth period. Our secondary analysis describes mortality, transfer out, and lost to follow-up (LTFU) as outcomes at age 15 years, using competing risk analysis. Among the 38,187 APHs included, 51% were female, 79% were from sub-Saharan Africa and 65% lived in low-income countries. APHs from 51 countries were included (Europe: 14 countries and 3,054 APHs; North America: 1 country and 1,032 APHs; South America and the Caribbean: 4 countries and 903 APHs; South and Southeast Asia: 7 countries and 2,902 APHs; sub-Saharan Africa, 25 countries and 30,296 APHs). Observation started as early as 1982 in Europe and 1996 in sub-Saharan Africa, and continued until at least 2014 in all regions. The median (interquartile range [IQR]) duration of adolescent follow-up was 3.1 (1.5–5.2) years for the total cohort and 6.4 (3.6–8.0) years in Europe, 3.7 (2.0–5.4) years in North America, 2.5 (1.2–4.4) years in South and Southeast Asia, 5.0 (2.7–7.5) years in South America and the Caribbean, and 2.1 (0.9–3.8) years in sub-Saharan Africa. Median (IQR) age at first visit differed substantially by region, ranging from 0.7 (0.3–2.1) years in North America to 7.1 (5.3–8.6) years in sub-Saharan Africa. The median age at ART start varied from 0.9 (0.4–2.6) years in North America to 7.9 (6.0–9.3) years in sub-Saharan Africa. The cumulative incidence estimates (95% confidence interval [CI]) at age 15 years for mortality, transfers out, and LTFU for all APHs were 2.6% (2.4%–2.8%), 15.6% (15.1%–16.0%), and 11.3% (10.9%–11.8%), respectively. Mortality was lowest in Europe (0.8% [0.5%–1.1%]) and highest in South America and the Caribbean (4.4% [3.1%–6.1%]). However, LTFU was lowest in South America and the Caribbean (4.8% [3.4%–6.7%]) and highest in sub-Saharan Africa (13.2% [12.6%–13.7%]). Study limitations include the high LTFU rate in sub-Saharan Africa, which could have affected the comparison of mortality across regions; inclusion of data only for APHs receiving ART from some countries; and unavailability of data from high-burden countries such as Nigeria.

**Conclusion:**

To our knowledge, our study represents the largest multiregional epidemiological analysis of APHs. Despite probable under-ascertained mortality, mortality in APHs remains substantially higher in sub-Saharan Africa, South and Southeast Asia, and South America and the Caribbean than in Europe. Collaborations such as CIPHER enable us to monitor current global temporal trends in outcomes over time to inform appropriate policy responses.

## Abstract 2

In an analysis of data on adolescents with HIV infection in 51 countries, Valériane Leroy and colleagues report on antiretroviral treatment and outcomes.

## Author summary

**Why was this study done?:**

- With increasing access to antiretroviral therapy across the globe, children with HIV acquired around the time of birth or through breastfeeding (perinatally acquired HIV) are surviving into adolescence. However, globally, adolescents living with perinatally acquired HIV experience poorer HIV-related outcomes compared to younger children and adults with HIV, dying more often and experiencing greater challenges in terms of treatment adherence and staying in care.
- A direct comparison of outcomes for adolescents living with perinatally acquired HIV across multiple regions of the world has not previously been conducted, yet such an understanding is required to inform the appropriate policy responses to meet the needs of this dynamic and complex population of adolescents.
- The Collaborative Initiative for Paediatric HIV Education and Research (CIPHER) global project describes for the first time, as far as we are aware, the global epidemiology of adolescents living with perinatally acquired HIV in terms of geographic and temporal trends and compares mortality, transfer, and loss to follow-up between 10 and 15 years of age across geographic regions, country income groups, and birth cohorts.

**What did the researchers do and find?:**

- Among 38,000 adolescents living with perinatally acquired HIV, 79% of whom were living in sub-Saharan Africa.
- Analysis by country income group suggested that patients in high-income countries (North America and Europe) had younger age, higher CD4 percent, and less impaired height when starting antiretroviral therapy compared to middle- or low-income countries.
- Lost to follow-up rate was lowest in South America and the Caribbean and highest in sub-Saharan Africa.
- HIV-associated mortality during adolescence was substantially higher in sub-Saharan Africa, South and Southeast Asia, and South America and the Caribbean than in Europe.

**What do these findings mean?:**

- Although the population of adolescents living with perinatally acquired HIV is likely to decline in the future due to declining new perinatally acquired HIV infections, there is still much work to be done to achieve equality in health and survival for all adolescents living with perinatally acquired HIV, irrespective of geographic location.
- Collaborations such as CIPHER are useful to monitor current global temporal trends in outcomes of adolescents living with perinatally acquired HIV over time to inform and guide policy responses.

## Introduction

It is estimated that almost 2.1 million (uncertainty bounds 1.4–2.7 million) adolescents aged 10–19 years are living with either perinatally or horizontally acquired HIV [1,2]. Prior to 2005, children perinatally infected with HIV in most of the world had poor access to antiretroviral therapy (ART), with high mortality during infancy and poor survival beyond childhood [3]. With the expansion of effective ART initially in Europe and North America, subsequently in South America and Asia, and now in Africa, the population of children living with perinatally acquired HIV surviving into adolescence and early adulthood is growing [1,4,5].

By the time children perinatally infected with HIV reach the developmental transition period of adolescence, they have been living for a decade with a chronic disease that even with ART treatment can still result in substantial morbidity [6]. Globally, it is recognized that adolescents living with perinatally acquired HIV (APHs) experience poorer HIV-related outcomes compared to younger children and adults, including higher mortality and virologic treatment failure rates and poorer retention in care [1,7–15]. Studies assessing the outcomes of APHs over time and across geographic and economic settings are limited [16]. Based on studies in adults, after 2 years on ART, HIV-associated mortality in South Africa approached that in the United States, and the differential between South Africa and Europe was substantially reduced [17]. As the global community pursues attainment of the Sustainable Development Goals by 2030, particularly to ensure healthy lives and promote wellbeing for all at all ages (Goal 3), to achieve gender equality (Goal 5), and to reduce inequality within and among countries (Goal 10), multiregional direct comparisons of APH outcomes can inform the appropriate policy responses to meet the needs of this dynamic and complex population of adolescents living with HIV [18].

The primary objective of this Collaborative Initiative for Paediatric HIV Education and Research (CIPHER) global project was to describe the global epidemiology of APH in terms of geographic and temporal trends of patient and treatment characteristics at entry into care, ART start, entry into adolescence (age 10 years), and last visit. Our secondary objective was to compare the outcomes of mortality, transfer out, and lost to follow-up (LTFU) between 10 and 15 years of age across regions, country income groups (CIGs), and birth cohorts.

## Methods

Primary data collection by all participating networks was approved by their respective research ethics boards of authority, and consent or assent for study participation was provided by participants as required. The pooling of data and analysis at the UCT data center was approved by the University of Cape Town Health Research Ethics Committee (UCT HREC reference 264/2014). The study concept and a priori analysis plan are available in supplementary material (S1 Analysis Plan). All analyses were prespecified.

### Study methods

The CIPHER Cohort Collaboration is a global network of observational pediatric HIV cohorts or cohort networks convened by CIPHER of the International AIDS Society. The following 12 cohort networks contributed data to this collaborative project: Baylor International Pediatric AIDS Initiative at Texas Children’s Hospital (BIPAI); European Pregnancy and Paediatric HIV Cohort Collaboration (EPPICC); International Epidemiology Databases to Evaluate AIDS (IeDEA)—Asia Pacific; IeDEA—Central Africa; IeDEA—East Africa; IeDEA—Southern Africa; IeDEA—West Africa; Caribbean, Central and South America Network for HIV Research (CCASAnet); Pediatric Late Outcomes Protocol (PACTG/IMPAACT 219/219c); Prospective Surveillance Study of Long-term Outcomes in HIV-infected Infants, Children and Adolescents (IMPAACT P1074); Médecins Sans Frontières (MSF) Pediatric Cohorts; Pediatric HIV/AIDS Cohort Study Adolescent Master Protocol (PHACS AMP); and Identifying Optimal Models for Care in Africa (Optimal Models-ICAP). The data contributed by the networks were drawn from a range of care settings, including dedicated research cohorts, routine care cohorts, and programmatic services. Using a standardized data transfer protocol based on the HIV Cohorts Data Exchange Protocol [19] and following quality checks and queries at the central University of Cape Town data center, individual-level data on 183,119 children infected with HIV were merged in May 2016. Participants contributed data to only a single network and there was no duplication of participants amongst networks.

### Analytic methods

We conducted a retrospective cohort analysis. APHs were defined as children infected with HIV with at least one documented HIV care visit prior to age 10 years, as a proxy for perinatally acquired HIV, and as having at least one additional HIV care visit after 10 years of age. Children with known nonvertical routes of HIV infection, e.g., horizontal transmission from blood products, unsafe injections, or sexual abuse, were excluded. Our primary analysis described patient and treatment characteristics of APH at key time points, including first HIV-associated clinic visit, ART start, age 10 years, and last visit, and compared these characteristics by geographic region, CIG, and birth cohort. Observation time was censored at 19 years of age in adolescents with follow-up beyond this age.

The first visit was defined as the first recorded date in the database of any contact with a healthcare facility for HIV-related care, and first visit measurements (height, CD4 T lymphocyte counts and percentages, and HIV viral load) were taken as the closest measurement to the first visit date but could be no later than 182 days after the first visit. If ART was started within 182 days of the first visit, only measurements up to 14 days after ART start were considered. The date of ART start was defined as the earliest date in the network database of initiation of any two antiretroviral drugs prior to the year 2000 or three or more antiretroviral drugs from the year 2000 or later. Measurements at ART start were taken as those closest to the ART start date but limited to a window of 182 days prior to or 14 days after ART start. Measurements at age 10 years were taken as those closest to the child’s 10th birthday, limited to a window of 182 days either side of the 10th birthday. The last visit was defined as the last date of any recorded visit, laboratory test, or ART record. Measurements at the last visit were taken as those closest to the last visit date, within a window of 18 months prior to the last visit date, to allow for only annual monitoring in stable patients on ART. It is possible for there to be overlap, with individual measurements classified as occurring at more than 1 time point, e.g., a measurement classified as a first visit measurement can also be classified as an ART start measurement and similarly for measurements at age 10 years and last visit. World Health Organization (WHO) height-for-age Z-scores (HAZs) were calculated for APHs in all regions from the measured heights using the WHO “igrowup_restricted” Stata macro for HAZ up to 5 years of age [20] and the “who2007” Stata macro for HAZ from 5–19 years of age [21]. Stunting was defined as HAZ < −2 standard deviations from the mean. Viral suppression was considered as an HIV viral load measurement of less than 400 copies/ml or less than the level of detection of the test at the time, if greater than 400 copies/ml. Geographic regions were categorized as Europe, North America, South America and the Caribbean, South and Southeast Asia, and sub-Saharan Africa. CIGs were assigned according to World Bank CIG classification for the median year of first visit for each country [22]. Birth cohorts were categorized as born prior to 1995, born between 1995 and 1999, and born between 2000 and 2005.

Our secondary analysis focused on patient outcomes between 10 and 15 years of age and classified as mortality, transfer out, LTFU, or alive and retained in care. Mortality included all-cause mortality, as reported in the database. Transfer out included documented transfer to a different HIV care site for any reason. LTFU was defined as no observed visit for more than 365 days before the last observed visit for the cohort. APHs classified as LTFU were censored 365 days after their last observed visit. APHs considered to be alive and in care at database closure were those not known to have died or transferred and with an observed visit within 365 days prior to the last visit for the cohort. Cumulative incidence functions for the outcomes mortality, transfer out, and LTFU at 15 years of age were calculated using competing risks analysis for the whole cohort as well as by region, CIG, and birth period, with person-time accruing from age 10 years [23]. Cumulative incidence functions for birth cohorts stratified separately by region or by CIG were calculated for the outcomes at 13 years of age due to few APHs in the most recent birth cohort having reached the age of 15 years. Transfer out and LTFU were both considered to be competing risks for mortality rather than censoring events. This approach was chosen because the survival distribution of adolescents transferred out or LTFU is likely to be different to those retained in care, with better survival in stable transferred patients and poorer survival in patients LTFU and possibly no longer on ART [23]. For comparison, cumulative mortality estimates at 15 years of age were also calculated using the Kaplan-Meier product limit estimator.

Mortality hazard ratios (HRs) and 95% confidence intervals (CIs) were calculated for geographic regions using Cox proportional hazard models, with Europe as the reference group. Continuous variables, including age, CD4 count, and CD4 percent were included in the Cox models as continuous variables after confirming a linear relationship with mortality. Proportionality assumptions were evaluated using the Schoenfeld test. Adjusted HRs (aHRs) were calculated, adjusting for baseline differences between regions. Missing CD4 and height measurements were imputed for the multivariable models using multiple imputation (MI) by chained equations [24]. The imputation model contained all measured variables and used predictive mean matching for CD4 counts. Imputation of missing CD4 measurements was performed for all countries and subsequently also restricted to only countries with at least 50 CD4 measurements at first visit or, for countries with fewer than 50 APHs, at least 50% of APHs with CD4 measurements at first visit. Sensitivity analyses were conducted to better understand how LTFU may have biased mortality estimates. Firstly, inverse probability weighting (IPW) was applied to the multivariable model, giving greater weight to APHs not LTFU but with characteristics similar to APHs who were LTFU. Secondly, under varying assumptions about the proportion of LTFU that could be due to mortality, mortality was randomly assigned to a proportion of APHs that were originally classified as LTFU [25]. Unadjusted HRs (uHRs) as well as cumulative incidence functions were recalculated under these assumptions. All analyses were conducted using Stata version 13.0 (StataCorp, College Station, TX, USA), and the “stcompet” package was used to calculate the cumulative incidence functions from the competing risks analysis. Figures were plotted using the ggplot2 package in R version 3.2.2 (R Foundation for Statistical Computing, Vienna, Austria).

## Results

**Fig 1:**
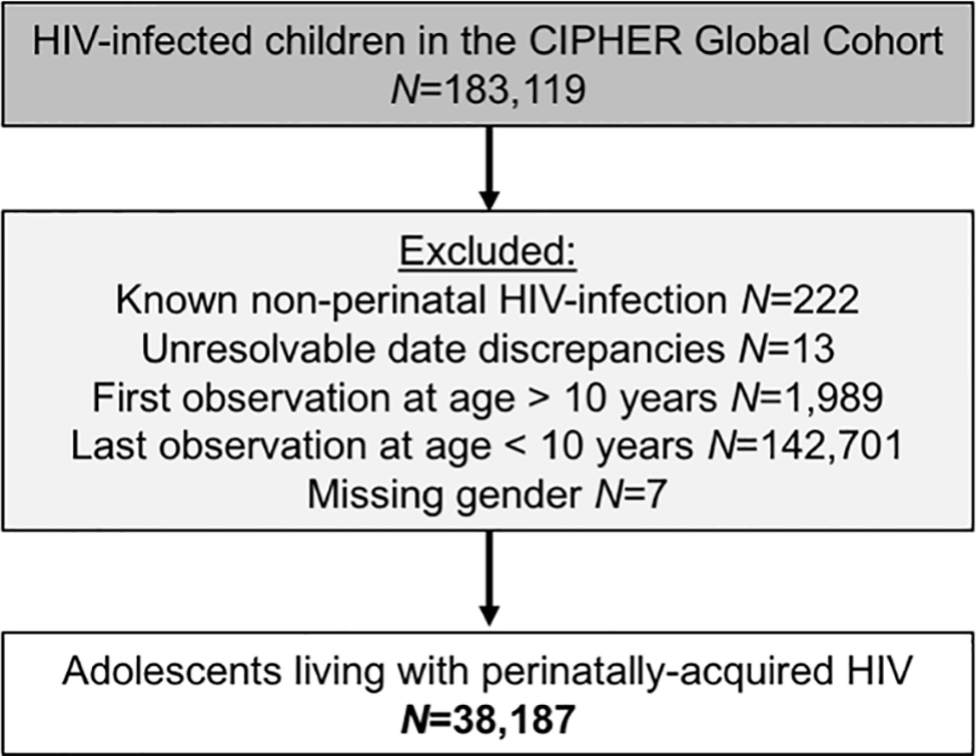
Flow diagram of inclusion of adolescents living with perinatally acquired HIV (*N* = 38,187). CIPHER, Collaborative Initiative for Paediatric HIV Education and Research.

Of the 183,119 children included in the CIPHER multiregional dataset, a total of 38,187 APHs were included (Fig 1), from 51 countries across 5 regions of the world, with 79% from sub-Saharan Africa (Table 1). Observation started as early as 1982 in Europe and 1996 in sub-Saharan Africa and continued until at least 2014 in all regions. The median (interquartile range [IQR]) year of birth was earliest in North America (1994 [1992–1996]) and latest in South and Southeast Asia, (2001 [1999–2002]). A total of 112,976 person-years were observed between 10 and 19 years of age, and the median (IQR) duration of adolescent follow-up was longest in Europe (6.4 [3.6–8.0] years) and shortest in sub-Saharan Africa (2.1 [0.9–3.8] years) (Table 1). Overall, 44% (46/104) of included cohorts provided data only on APHs that had ever received ART.

**Table 1:** Profile of geographic regions included in the CIPHER global cohort adolescent analysis (*N* = 38,187 adolescents).

Region	Countries included	Number of study centers	Number of adolescents (%)	Observation period	Year of birth median (IQR)	Duration of adolescent follow-up (from age 10 years) median (IQR) years
**Europe**	Belgium, France, Ireland, Italy, Netherlands, Poland, Portugal, Romania, Russian Federation, Spain, Sweden, Switzerland, Ukraine, United Kingdom	153	3,054 (8.0)	1982–2015	1995 (1991–1999)	6.4 (3.6–8.0)
**North America**	United States of America	117	1,032 (2.7)	1991–2014	1994 (1992–1996)	3.7 (2.0–5.4)
**South and Southeast Asia**	Cambodia, India, Indonesia, Malaysia, Myanmar, Thailand, Vietnam	73	2,902 (7.6)	1994–2014	2001 (1999–2002)	2.5 (1.2–4.4)
**South America and the Caribbean**	Argentina, Brazil, Haiti, Honduras	6	903 (2.4)	1990–2015	1998 (1995–2000)	5.0 (2.7–7.5)
**Sub-Saharan Africa**	Benin, Botswana, Burkina Faso, Burundi, Cameroon, Central African Republic, Democratic Republic of Congo, Côte d’Ivoire, Ethiopia, Ghana, Guinea, Kenya, Lesotho, Malawi, Mali, Mozambique, Rwanda, Senegal, South Africa, Swaziland, Tanzania, Togo, Uganda, Zambia, Zimbabwe	565	30,296 (79.3)	1996–2015	2000 (1999–2002)	2.1 (0.9–3.8)
**Total**		914	38,187	1982–2015	2000 (1998–2002)	3.1 (1.5–5.2)

Abbreviations: CIPHER, Collaborative Initiative for Paediatric HIV Education and Research; IQR, interquartile range.

### Comparison by geographic region

**Fig 2:**
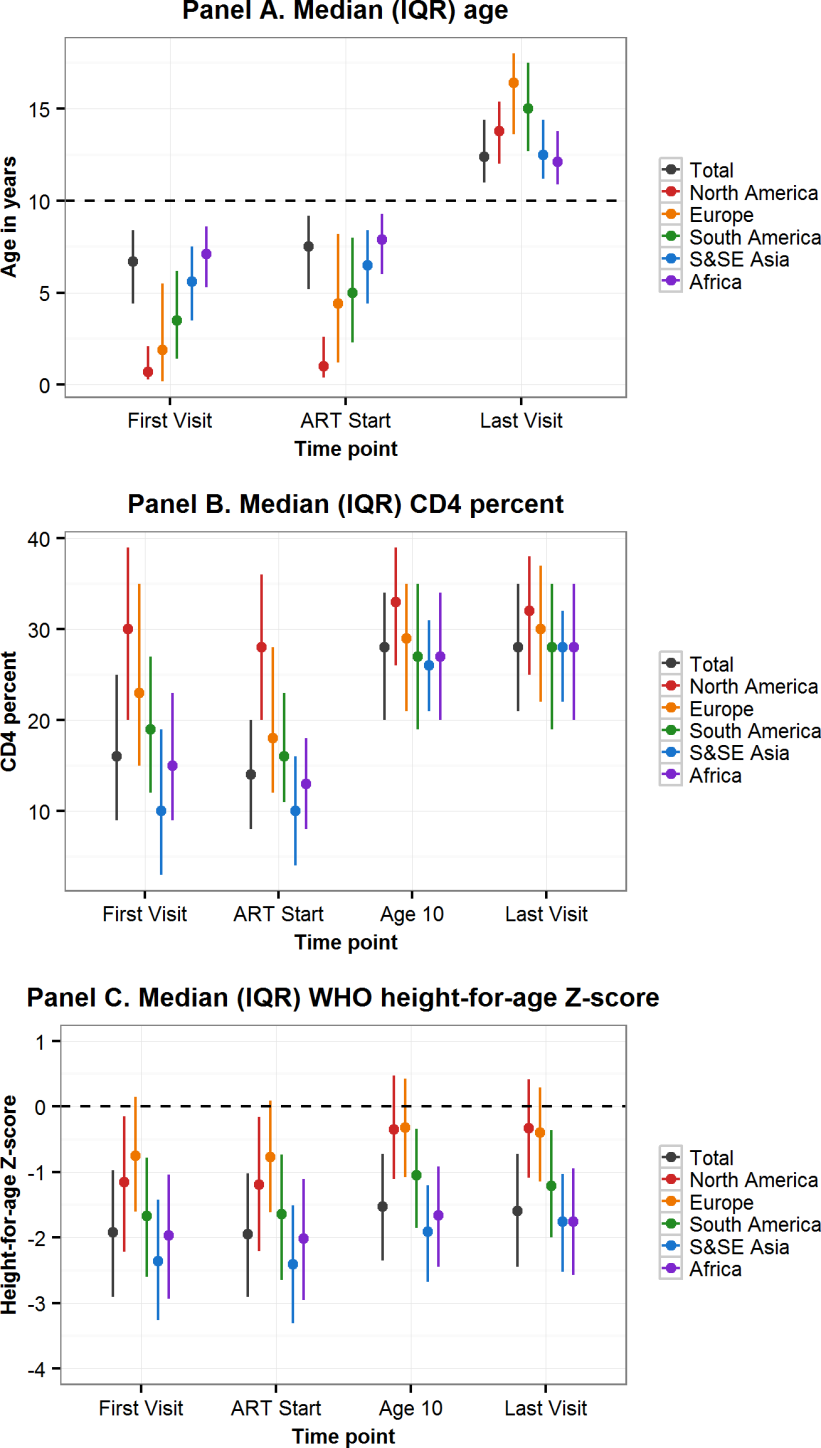
Comparison by geographic region of characteristics at first visit, ART start, age 10 years, and last visit of adolescents living with perinatally acquired HIV. ART, antiretroviral therapy; IQR, interquartile range; S&SE Asia, South and Southeast Asia; WHO, World Health Organization.

More than two-thirds of APHs living in sub-Saharan Africa and South and Southeast Asia were born between 2000 and 2005, compared to only 7% of APHs in North America (Table 2). In all regions, approximately half of the APHs were female. Median (IQR) age at first visit differed substantially by region, ranging from 0.7 (0.3–2.1) years in North America to 7.1 (5.3–8.6) years in sub-Saharan Africa. Similarly, APHs in North America started ART at a median (IQR) age of 0.9 (0.4–2.6) years, compared to 7.9 (6.0–9.3) years in sub-Saharan Africa (Table 2, Fig 2 Panel A). In the 61% of APHs with a CD4 count or percent measurement recorded at ART start, the median (IQR) CD4 count was 321 (165–575), with substantial variation by region (Table 2). Similarly, median (IQR) CD4 percent at ART start varied by region and was highest in North America (28% [20%–36%]) and lowest in South and Southeast Asia (10% [4%–16%]). By age 10 years and last visit there was less variation in CD4 percent by region (Fig 2 Panel B). Median (IQR) HAZ at first visit and ART start was well below WHO normative data in all regions and lowest in South and Southeast Asia, at −2.36 (−3.26–−1.42) at first visit and −2.41 (−3.31–−1.51) at ART start (Fig 2 Panel C). In APHs in Europe and North America, HAZ improved by age 10 years and last visit, but at least 25% of APHs in South and Southeast Asia, South America and the Caribbean, and sub-Saharan Africa remained stunted at their last visit (Table 2). Eighty-eight percent of APHs received ART at some stage, of whom 12% started ART after age 10 years and 80% remained on ART at their last visit. Of the 7,401 (19%) APHs not known to be on ART at their last visit, 62% were ART naïve. Only 38% of all APHs had an HIV viral load recorded at some stage, and this proportion was lowest in sub-Saharan Africa, at 25% (Table 2). Of those with an HIV viral load measurement and on ART at their last visit, 72% (9,388/13,114) were virologically suppressed.

**Table 2:** Adolescent characteristics at first visit, ART start, age 10 years, and last visit and cumulative incidence of outcomes (mortality, transferred out, LTFU), compared by region.

Characteristic	Total	Europe	North America	South and Southeast Asia	South America and the Caribbean	Sub-Saharan Africa
**Total *N*** (row %)	**38,187 (100)**	**3,054 (8.0)**	**1,032 (2.7)**	**2,902 (7.6)**	**903 (2.4)**	**30,296 (79.3)**
Birth Cohort, ***N*** (%)						
Pre-1995	**2,660 (7.0)**	**1,399 (45.8)**	**640 (62.0)**	**91 (3.1)**	**182 (20.2)**	**348 (1.1)**
1995–1999	**13,267 (34.7)**	**989 (32.4)**	**318 (30.8)**	**918 (31.6)**	**446 (51.6)**	**10,596 (35.0)**
2000–2005	**22,260 (58.3)**	**666 (21.8)**	**74 (7.2)**	**1,893 (65.2)**	**275 (30.5)**	**19,352 (63.9)**
Male, *N* (%)	**18,863 (49.4)**	**1,475 (48.3)**	**515 (49.9)**	**1,449 (49.9)**	**417 (46.2)**	**15,007 (49.5)**
**Age in years**, median (IQR)						
First visit	**6.7 (4.4–8.4)**	**1.9 (0.2–5.5)**	**0.7 (0.3–2.1)**	**5.6 (3.5–7.5)**	**3.5 (1.4–6.2)**	**7.1 (5.3–8.6)**
ART start	**7.5 (5.2–9.2)**	**4.4 (1.2–8.2)**	**0.9 (0.4–2.6)**	**6.5 (4.4–8.4)**	**5.0 (2.2–8.0)**	**7.9 (6.0–9.3)**
Last visit	**12.4 (11.1–14.4)**	**16.4 (13.6–18.0)**	**13.7 (12.0–15.4)**	**12.5 (11.2–14.4)**	**15.0 (12.7–17.5)**	**12.1 (10.9–13.8)**
**CD4 count in cells/mm^3^**, median (IQR)						
First visit all ages (***N*** = 19,979)	**427 (200–757)**	**768 (375–1,580)**	**1,263 (775–2,207)**	**252 (68–575)**	**583 (268–990)**	**405 (201–699)**
First visit if age ≥5 years (***N*** = 14,585)	**358 (165–632)**	**415 (202–629)**	**504 (298–598)**	**189 (53–447)**	**349 (120–562)**	**370 (180–646)**
ART start all ages (***N*** = 20,608)	**321 (165–575)**	**464 (241–1,029)**	**1,129 (702–1,921)**	**219 (73–406)**	**462 (222–785)**	**310 (165–520)**
ART start if age ≥5 years (***N*** = 16,612)	**292 (161–469)**	**298 (161–469)**	**590 (384–767)**	**189 (60–322)**	**328 (139–540)**	**301 (158–500)**
Age 10 years (***N*** = 26,953)	**685 (445–972)**	**697 (468–970)**	**796 (575–1,049)**	**746 (491–999)**	**639 (412–914)**	**671 (430–964)**
Last visit (***N*** = 31,951)	**687 (464–946)**	**628 (434–868)**	**700 (494–930)**	**749 (541–1,004)**	**601 (381–861)**	**689 (460–953)**
**CD4%**, median (IQR)						
First visit (***N*** = 13,674)	**16 (9–25)**	**23 (15–35)**	**30 (20–39)**	**10 (3–19)**	**19 (12–27)**	**15 (9–23)**
**ART start** (***N*** = 14,740)	**14 (8–20)**	**18 (12–28)**	**28 (20–36)**	**10 (4–16)**	**16 (11–23)**	**13 (8–18)**
Age 10 years (***N*** = 17,974)	**28 (20–34)**	**29 (21–35)**	**33 (26–39)**	**26 (21–31)**	**27 (19–35)**	**27 (20–34)**
Last visit (***N*** = 23,292)	**29 (21–35)**	**30 (22–37)**	**32 (25–38)**	**28 (22–32)**	**28 (19–35)**	**28 (20–35)**
**HAZ**, median (IQR)						
First visit (***N*** = 20,269)	**−1.92 (−2.91–−0.97)**	**−0.75 (−1.60–0.15)**	**−1.15 (−2.22–−0.15)**	**−2.36 (−3.26–−1.42)**	**−1.67 (−2.60–−0.78)**	**−1.97 (−2.94–−1.04)**
ART start (***N*** = 20,372)	**−1.95 (−2.91–−1.02)**	**−0.77 (−1.61–0.09)**	**−1.19 (−2.21–−0.16)**	**−2.41 (−3.31–−1.51)**	**−1.64 (−2.65–−0.73)**	**−2.02 (−2.95–−1.11)**
Age 10 years (***N*** = 26,883)	**−1.53 (−2.35–−0.72)**	**−0.32 (−1.08–0.43)**	**−0.35 (−1.11–0.47)**	**−1.91 (−2.68–−1.20)**	**−1.05 (−1.85–−0.34)**	**−1.66 (−2.45–−0.91)**
Last visit (***N*** = 32,752)	**−1.59 (−2.45–−0.72)**	**−0.40 (−1.14–0.29)**	**−0.33 (−1.09–0.42)**	**−1.76 (−2.52–−1.03)**	**−1.21 (−2.00–−0.36)**	**−1.75 (−2.57–−0.94)**
ART, *N* (%)						
Ever received	**33,514 (87.8)**	**2,889 (94.6)**	**1,016 (98.5)**	**2,708 (93.3)**	**883 (97.8)**	**26,018 (85.9)**
Started > age 10 years	**4,037 (12.0)**	**371 (12.8)**	**1 (0.1)**	**217 (8.0)**	**96 (10.9)**	**3,352 (12.9)**
On ART at age 10 years	**25,713 (67.3)**	**2,021 (66.2)**	**866 (83.9)**	**2,400 (82.7)**	**697 (77.2)**	**19,729 (65.1)**
On ART at last visit	**30,072(80.3)**	**2,540 (84.1)**	**878 (86.1)**	**2,565 (89.8)**	**768 (89.8)**	**23,321 (78.5)**
**Virologic suppression**, n/N (%)						
Age 10 years	**6,919/10,209 (67.8)**	**1,442/2,500 (57.7)**	**576/1,012 (56.9)**	**1,099/1,332 (82.5)**	**267/515 (51.8)**	**3,535/4,850 (72.9)**
Last visit	**9,741/14,200 (68.6)**	**2,149/2,994 (71.8)**	**617/1,020 (60.5)**	**1,544/1,882 (82.0)**	**403/601 (67.1)**	**5,028/7,703 (65.3)**
**Cumulative incidence (95% CI) at age 15 years**						
Mortality (%)	**2.6 (2.4–2.8)**	**0.8 (0.5–1.2)**	**1.1 (0.5–2.1)**	**2.7 (1.9–3.8)**	**4.4 (3.1–6.1)**	**2.9 (2.7–3.2)**
Transferred out (%)	**15.6 (15.1–16.0)**	**3.5 (2.9–4.3)**	**1.9 (1.1–3.1)**	**6.7 (5.5–8.0)**	**6.5 (4.9–8.5)**	**19.3 (18.7–20.0)**
Lost to follow-up (%)	**11.3 (10.9–11.8)**	**6.1 (5.2–7.0)**	**8.9 (6.7–11.3)**	**7.1 (5.6–8.7)**	**4.8 (3.4–6.7)**	**13.2 (12.6–13.7)**

Note: 48.5%, 47.7%, and 26.9% of first visit CD4 count, CD4 percent, and HAZ measurements, respectively, overlapped with ART start measurements, and 14.9%, 15.5%, and 7.3% of age 10 year CD4 count, CD4 percent, and HAZ measurements, respectively, overlapped with last visit measurements.

Abbreviations: ART, antiretroviral therapy; CI, confidence interval; HAZ, WHO height-for-age Z-score; IQR, interquartile range; LTFU, loss to follow-up.

By competing risks analysis, the cumulative incidence estimates (95% CI) at 15 years of age for mortality, transfers out, and LTFU for all APHs were 2.6% (2.4%–2.8%), 15.6% (15.1%–16.0%), and 11.3% (10.9%–11.8%), respectively (Table 2). Cumulative incidence of mortality before any other competing event occurred was estimated to be lowest in Europe (0.8% [0.5%–1.1%]) and highest in South America and the Caribbean (4.4% [3.1%–6.1%]) (Table 2). However, LTFU before mortality or transfer out was lowest in South America and the Caribbean (4.8% [3.4%–6.7%]) and highest in sub-Saharan Africa (13.2% [12.6%–13.7%]). Transfers out before mortality or LTFU were also highest in sub-Saharan Africa (19.3% [18.7%–20.0%]). Cumulative mortality (95% CI) when estimated by the Kaplan-Meier product limit estimator was similar, at 3.0% (2.8%–3.3%) for the total cohort, ranging from 0.8% (0.5%–1.2%) in Europe to 4.7% (3.3%–6.6%) in South America and the Caribbean (S1 Table).

### Comparison by CIG

**Fig 3:**
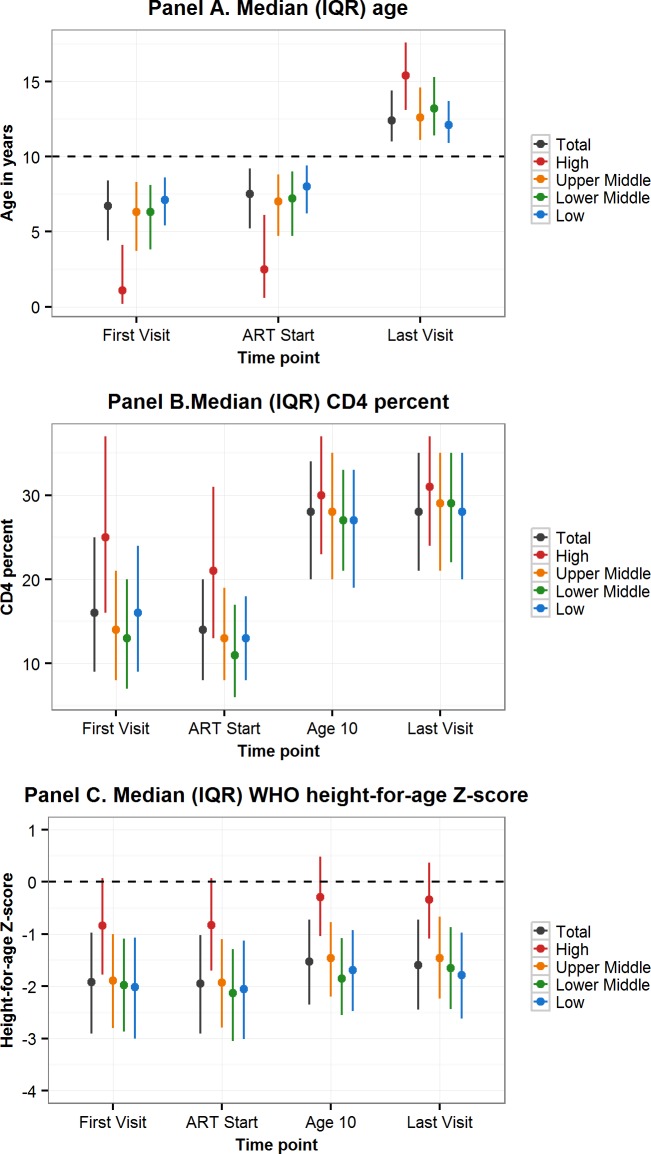
Comparison by CIG of characteristics at first visit, ART start, age 10 years, and last visit of adolescents living with perinatally acquired HIV. ART, antiretroviral therapy; CIG, country income group; IQR, interquartile range; WHO, World Health Organization.

Sixty-five percent of all APHs in this cohort lived in low-income countries, 7.9% in lower-middle-income-, 17.5% upper-middle-income-, and 9.7% in high-income countries. Variation in characteristics by CIG followed the geographic region trends, with younger age, higher CD4 percent, and less impaired HAZ at first visit and ART start in APHs in high-income countries compared to upper-middle-, lower-middle-, or low-income countries (Table 3, Fig 3). Mortality before transfer out or LTFU was lowest in high-income countries (0.9% [0.6%–1.3%]) and highest in low-income countries (3.5% [3.1%–3.8%]) (Table 3). However, LTFU before mortality or transfer out was highest in upper-middle-income countries (12.8% [11.8%–13.9%]).

**Table 3:** Adolescent characteristics at first visit, ART start, age 10 years, and last visit and cumulative incidence of outcomes (mortality, transferred out, LTFU), compared by CIG.

Characteristic	Total	Low Income	Lower-Middle Income	Upper-Middle Income	High Income
**Total *N*** (row %)	38,187 (100)	24,794 (64.9)	3,015 (7.9)	6,669 (17.5)	3,709 (9.7)
**Male, *N*** (%)	18,863 (49.4)	12,191 (49.2)	1,503 (49.9)	3,372 (50.6)	1,797 (48.5)
**Age in years, median (IQR)**					
First visit	6.7 (4.4–8.4)	7.1 (5.4–8.6)	6.3 (3.8–8.1)	6.3 (3.7–8.3)	1.1 (0.2–4.1)
ART start	7.5 (5.2–9.2)	8.0 (6.2–9.4)	7.2 (4.7–9.0)	7.0 (4.7–8.8)	2.5 (0.6–6.1)
Last visit	12.4 (11.1–14.4)	12.1 (10.9–13.7)	13.2 (11.4–15.3)	12.6 (11.1–14.6)	15.4 (13.1–17.6)
**CD4 count in cells/mm^3^, median (IQR)**					
First visit (***N*** = 19,979)	427 (200–757)	405 (194–707)	348 (142–610)	393 (183–709)	924 (470–1,800)
First visit if age ≥5 years (***N*** = 14,585)	358 (165–632)	377 (176–664)	302 (116–540)	298 (132–527)	442 (234–660)
ART start (***N*** = 20,608)	321 (165–575)	302 (155–504)	277 (108–455)	336 (171–602)	624 (294–1,318)
ART start if age ≥5 years (***N*** = 16,612)	292 (161–469)	300 (154–504)	245 (91–360)	283 (139–478)	320 (190–502)
Age 10 years (***N*** = 26,953)	685 (445–972)	661 (421–955)	702 (463–953)	719 (472–1,004)	730 (500–998)
Last visit (***N*** = 31,951)	687 (464–946)	679 (443–956)	692 (492–917)	727 (511–968)	650 (451–882)
**CD4%, median (IQR)**					
First visit (***N*** = 13,674)	16 (9–25)	16 (9–24)	13 (7–20)	14 (8–21)	25 (16–37)
ART start (***N*** = 14,740)	14 (8–20)	13 (8–18)	11 (6–17)	13 (8–19)	21 (13–31)
Age 10 years (***N*** = 17,974)	28 (20–34)	27 (19–34)	27 (21–33)	28 (20–35)	30 (23–37)
Last visit (***N*** = 23,292)	29 (21–35)	28 (20–35)	29 (22–35)	29 (22–35)	31 (24–37)
**HAZ, median (IQR)**					
First visit (***N*** = 20,269)	−1.92 (−2.91–−0.97)	−2.02 (−3.00–−1.07)	−1.98 (−2.87–−1.09)	−1.89 (−2.80–−1.00)	−0.84 (−1.78–0.07)
ART start (***N*** = 20,372)	−1.95 (−2.91–−1.02)	−2.05 (−3.01–−1.13)	−2.13 (−3.05–−1.29)	−1.93 (−2.79–−1.10)	−0.83 (−1.70–0.07)
Age 10 years (***N*** = 26,883)	−1.53 (−2.35–−0.72)	−1.69 (−2.48–−0.92)	−1.83 (−2.55–−1.08)	−1.46 (−2.20–−0.77)	−0.29 (−1.04–0.48)
Last visit (***N*** = 32,752)	−1.59 (−2.45–−0.72)	−1.79 (−2.62–−0.97)	−1.65 (−2.44–−0.87)	−1.46 (−2.24–−0.67)	−0.34 (−1.09–0.37)
**ART, *N* (%)**					
Ever received	33,514 (87.8)	20,851 (84.1)	2,776 (92.1)	6,351 (95.2)	3,536 (95.3)
Started > age 10 years	4,037 (12.0)	3,028 (14.5)	305 (11.0)	423 (6.7)	281 (7.9)
On ART at age 10 years	25,713 (67.3)	15,444 (62.3)	2,279 (75.6)	5,323 (79.8)	2,667 (71.9)
On ART at last visit	30,072 (80.3)	18,408 (75.9)	2,589 (87.8)	6,002 (91.1)	3,073 (83.8)
**Virologic suppression, n/N (%)**					
Age 10 years	6,919/10,209 (67.8)	467/932 (50.2)	885/1,039 (85.2)	3,637/4,843 (75.1)	1,930/3,396 (56.8)
Last visit	9,741/14,200 (68.6)	1,342/2,777 (48.3)	1,210/1,486 (81.4)	4,684/6,274 (74.7)	2,505/3,663 (68.4)
**Cumulative incidence (95% CI) at age 15 years**					
Mortality (%)	2.6 (2.4–2.8)	3.5 (3.1–3.8)	2.7 (2.1–3.5)	1.4 (1.1–1.9)	0.9 (0.6–1.3)
Transferred out (%)	15.6 (15.1–16.0)	16.7 (16.1–17.4)	14.3 (12.8–15.8)	2.1 (2.0–2.3)	3.5 (2.9–4.2)
Lost to follow-up (%)	11.3 (10.9–11.8)	12.6 (12.0–13.2)	7.5 (6.3–8.8)	12.8 (11.8–13.9)	6.7 (5.8–7.7)

Abbreviations: ART, antiretroviral therapy; CI, confidence interval; CIG, country income group; HAZ, height-for-age-Z-score; IQR, interquartile range; LTFU, lost to follow-up.

### Comparison by birth cohort

Fifty-eight percent of all APHs were born in the year 2000 or later, ranging from 0.3% living in North America and 2.8% in high-income countries to 86.9% living in sub-Saharan Africa and 73.6% in low-income countries (S2 Table). Three-quarters (76.7%) of APHs born prior to 1995 lived in Europe and North America, and age at first visit and ART start appeared to be younger and CD4 count, CD4 percent, and HAZ at first visit and ART start appeared to be better in this group compared to those born in later calendar periods (S2 Table, S1 Fig). However, for the two more recent birth cohorts, the majority of APHs were from sub-Saharan Africa, and age at first visit and ART start was younger and CD4 count higher in APHs born during 2000–2005 than 1995–1999. HAZ, however, did not show any improvement over time at ART start or at last visit for APHs born between 2000 and 2005 compared to APHs born between 1995 and 1999. Mortality before transfer or LTFU was lowest in APHs born between 2000 and 2005 (1.84% [95% CI 1.50%–2.23%]), although LTFU in this group was also more than double that of the previous 2 birth periods (23.34% [95% CI 19.96%–26.88%]). When looking at birth cohort trends by region, mortality declined in every region for APHs born between 2000 and 2005 compared to those born in the earlier birth cohorts, and no mortality was observed in the most recent APH birth cohort in Europe, North America, and South America and the Caribbean (S3 Table). However, LTFU increased for APHs born between 2000 and 2005 in all regions except South America and the Caribbean.

### Mortality hazards compared by region

Relative to Europe, the unadjusted mortality HR (95% CI) was significantly higher in South and Southeast Asia (3.21 [2.03–5.07]), South America and the Caribbean (6.07 [3.87–9.50]), and sub-Saharan Africa (4.35 [3.02–6.28]) but not in North America (1.70 [0.87–3.31]) (Table 4, model 1). After controlling for baseline characteristics, including sex, birth cohort, age at first visit, and any ART received, the aHR increased slightly for North America and decreased for sub-Saharan Africa (Table 4, model 2). Inclusion of IPW in the model marginally reduced the HR further for all regions except North America (Table 4, model 3). Adjustment for CD4 measures, either as CD4 count or CD4 percent, at first visit only or time-updated and with or without MI for missing CD4 measures, also altered the individual region aHRs relative to Europe, but the general pattern remained of elevated mortality in all regions relative to Europe and substantially elevated mortality in sub-Saharan Africa and South America and the Caribbean (Table 4 and S4 Table).

**Table 4:** Mortality HRs (95% CIs) by region with reference to Europe.

North America	South and Southeast Asia	South America and the Caribbean	Sub-Saharan Africa
**1. Crude mortality HR compared to Europe (*N* = 38,187)**			
1.70 (0.87–3.31)	3.21 (2.03–5.07)	6.07 (3.87–9.50)	4.35 (3.02–6.28)
**2. Adjusted for complete baseline characteristics^a^ (*N* = 38,187)**			
2.03 (1.02–4.02)	2.96 (1.79–4.88)	5.94 (3.77–9.38)	3.37 (2.17–5.23)
**3. Adjusted for complete baseline characteristics^a^ with IPW (*N* = 38,187)**			
2.52 (1.24–5.11)	2.78 (1.75–4.42)	5.61 (3.58–8.77)	3.23 (2.21–4.73)
**4. Adjusted for baseline characteristics^a^, including CD4 percent, using complete cases only (*N* = 13,699)**			
1.84 (0.58–5.81)	1.90 (0.73–4.91)	4.20 (1.72–10.29)	4.09 (1.84–9.13)
**5. Adjusted for baseline characteristics^a^, including CD4 percent, with imputation for missing CD4 (*N* = 38,187)**			
2.34 (1.19–4.71)	1.87 (1.12–3.13)	5.00 (3.17–7.90)	2.70 (1.75–4.19)
**6. Adjusted for baseline characteristics^a^, including CD4 percent, with restricted imputation^b^ (*N* = 33,126)**			
1.97 (0.97–4.02)	1.56 (0.91–2.67)	4.16 (2.54–6.80)	2.42 (1.51–3.87)
**7. Adjusted for baseline characteristics^a^, including CD4 count, using complete cases only (*N* = 19,979)**			
2.18 (0.71–6.66)	3.16 (1.44–6.96)	5.52 (2.62–11.67)	4.57 (2.23–9.35)
**8. Adjusted for baseline characteristics^a^, including CD4 count, with imputation (*N* = 38,187)**			
2.37 (1.19–4.71)	2.40 (1.45–3.96)	5.49 (3.48–8.65)	3.01 (1.95–4.67)
**9. Adjusted for baseline characteristics^a^, including CD4 count, with restricted imputation^b^ (*N* = 33,126)**			
2.02 (0.99–4.11)	2.05 (1.20–3.49)	4.67 (2.86–7.63)	2.70 (1.68–4.33)
**10. Adjusted for baseline characteristics^a^ and time-updated CD4 count, with imputation (*N* = 38,187)**			
2.07 (1.05–4.10)	4.06 (2.45–6.73)	5.49 (3.46–8.71)	3.58 (2.28–5.64)
**11. Adjusted for baseline characteristics^a^, including CD4 count and HAZ, with imputation (*N* = 38,187)**			
2.31 (1.16–4.60)	2.21 (1.33–3.67)	5.21 (3.29–8.23)	2.85 (1.83–4.43)

^a^Sex, birth period (pre-1995, 1995–1999, 2000–2005), age at first visit, antiretroviral therapy (never started, started on dual therapy before 2000, started on triple therapy).

^b^Multiple imputation for missing CD4 restricted to countries with at least 50 CD4 observations at first visit, or <50% missing CD4 if total country *N* < 50.

Abbreviations: HAZ, height-for-age-Z-score; HR, hazard ratio; IPW, inverse probability weights.

### Sensitivity analyses

In sensitivity analyses, under varying assumptions of the proportion of APHs classified as LTFU who may be cases of unascertained mortality, the cumulative incidence for mortality before transfer out or LTFU in sub-Saharan Africa could be as high as 14.9% (95% CI 14.3%–15.5%) if 100% of APHs LTFU was truly mortality (S5 Table). Under the assumption that 100% of LTFU is due to mortality in all regions, the relative difference in mortality is attenuated in all regions in comparison to Europe, with the highest uHR in sub-Saharan Africa, at 1.46 (95% CI 1.33–1.61) (S6 Table). If it is differentially assumed that, in sub-Saharan Africa, 50% of LTFU is unascertained mortality but only 5% in all other regions, the uHR for mortality in sub-Saharan Africa may be as high as 7.09 (95% CI 5.45–9.23), relative to Europe.

## Discussion

To our knowledge, this is the largest analysis of APHs to date, describing the characteristics and outcomes of more than 38,000 APHs across more than 100,000 person-years of follow-up during adolescence, from 5 regions of the world, including 14 of the 15 highest adolescent HIV burden countries [1] and across low-, middle-, and high-income countries. This analysis essentially describes characteristics of and care for younger adolescents (10–14 years of age), 79% of whom were living in sub-Saharan Africa. Our findings show that APHs in North America and Europe, as well as high-income group countries, generally presented to care and started ART at a younger age and with higher CD4 counts, and less impaired height, compared to other regions or CIGs. Conversely, age at presentation to care and ART start was highest in sub-Saharan Africa. Despite probable under-ascertained mortality in some regions, the hazard of HIV-associated mortality during adolescence was substantially higher in sub-Saharan Africa, South and Southeast Asia, and South America and the Caribbean than in Europe. Results suggested that mortality may also have been higher in North America than in Europe. Analysis by CIG followed these geographic trends, with results suggesting younger age, higher CD4 percent, and less impaired height at first visit and ART start in high-income countries compared to middle- or low-income countries.

Results also suggested a marked difference in regional and income group distributions across birth cohort groups. APHs from North America and Europe and, likewise, high-income countries predominated in the earliest birth cohort, with minimal representation from Asia and Africa, while the most recent birth cohort was dominated by APHs living in sub-Saharan Africa. Younger age, higher CD4, and less impaired growth in the earliest birth cohort reflected this regional and CIG distribution. These vastly different characteristics of APHs across regions and over time require careful adjustment and interpretation due to different effects of variables across time. Nevertheless, some improvements for all APHs born after 2000 compared to those born during 1995–1999 are evident, including younger age and higher CD4 count at first presentation and ART start. This trend is expected, as criteria for initiating ART have progressively become less restrictive, with higher or no CD4 thresholds, and access to ART has expanded across the globe [3,26–29]. Despite this, height was still severely impacted in APHs in the most recent birth cohort. This may reflect that, although APHs born during 2000–2005 started ART earlier than APHs born during 1995–1999, they still only started at a median of 7 years of age, having missed the benefits of early ART on growth and probably also cognition and other morbidities, although the latter two outcomes were not evaluated in this analysis [30–32].

Comparing mortality across the regions is limited by high LTFU in sub-Saharan Africa relative to other regions. Furthermore, LTFU rates in Europe may be overestimated due to a reporting delay for some cohorts. Higher LTFU has been described in cohorts with shorter durations of follow-up, in which people may not yet have had the opportunity to return to care, while in cohorts of longer durations, people previously considered LTFU subsequently return to care [33]. This could affect patients born in the most recent birth cohorts in our analysis, particularly in sub-Saharan Africa. Methods have advanced in adult HIV cohort research to informatively adjust mortality estimates for under-ascertainment in those LTFU, informed by studies that actively traced patients LTFU to determine their vital status [34–37]. However, a recent systematic review identified few such tracing studies in children or adolescents living with HIV [25]. One study from Malawi traced 201 children who were LTFU and, of the 79% who were successfully traced, 11% had died, 26% had transferred to another clinic, and 25% were alive but no longer on ART [38]. A better understanding of how program LTFU can bias mortality estimates specifically in adolescents is needed to truly understand the impact on mortality in adolescents living with HIV [1].

This study has limitations. As mode of transmission is generally poorly captured in routine care cohorts, we used a pragmatic definition of APH and included only children infected with HIV who had entered care before age 10 years. This was done to ensure exclusion of adolescents with horizontally acquired HIV, who have a very different disease profile during adolescence to APHs [39]. As a result of this approach, we may have excluded a relatively small but important group of APHs diagnosed and entering care after age 10 years [40,41]. Our analysis does not include Nigeria, a country with the second largest burden of adolescents living with HIV and the only country in which mortality in younger adolescents (10–14 years of age) is estimated to be rising [1]. Furthermore, the North American region is represented only by the US in this analysis, and findings may not necessarily be generalizable to other North American countries. Our analysis may overrepresent APHs treated in healthcare settings with higher standards of care compared to the general population of adolescents, thus underestimating true mortality in APHs. Additionally, approximately 44% of included cohorts collect data only on children started on ART; thus, the proportion on ART observed in this analysis is likely overestimated for some regions. HIV viral load measurements were sparsely available and possibly selectively performed in the lower CIGs, with likely targeting of HIV viral load measurements to children with clinical or immunological failure, in which HIV viral load testing is not part of routine monitoring. This would result in overestimation of the proportion of patients with an unsuppressed HIV viral load, and these HIV viral load data should be interpreted with care in this analysis.

The current generation of APHs, and those represented in this CIPHER analysis, largely reflect children infected with HIV who survived early childhood without ART but at the same time experienced substantial growth morbidity and possibly other morbidities not measured in this analysis. This current generation may be substantially different to future cohorts of APHs, who will have been more likely to have started ART in infancy and may be affected by different issues. It is expected that APH survival will continue to improve with greater access to early infant diagnosis and universal ART for all people living with HIV [42]. Although the population of APHs is likely to decline in the future due to declines in new perinatally acquired HIV infections, there is still a lot of work to be done to achieve equality in health and survival for all APHs, irrespective of geographic location. Collaborations such as CIPHER enable us to monitor current global temporal trends in APH outcomes over time to ensure that this and future generations of APHs across the globe have the potential to thrive and contribute to society, outcomes that were denied to previous generations of children infected with HIV.

In summary, our analysis of a large cohort of APHs between 1982 and 2014 across several regions of the globe suggests that APHs generally entered HIV care at an earlier age in high-income countries compared to other CIGs. Despite probable under-ascertainment, mortality continued to be substantially higher in sub-Saharan Africa, South and Southeast Asia, and South America and the Caribbean than in Europe and warrants further monitoring and understanding.

## Supporting information

S1 STROBE Checklist STROBE checklist. (DOC)

S1 Text Group authorship list. (DOCX)

S2 Text Acknowledgments. (DOCX)

S1 Analysis Plan Project concept analysis plan. (PDF)

S1 Table Standard survival mortality estimates. (DOCX)

S2 Table Characteristics by birth cohort. (DOCX)

S3 Table Cumulative incidence 10–13 sensitivity. (DOCX)

S4 Table Selected full models. (DOCX)

S5 Table Cumulative incidence 10–15 sensitivity. (DOCX)

S6 Table Crude mortality hazard ratios sensitivity. (DOCX)

S1 Fig Comparison by birth cohort. (TIFF)

## Abbreviations

aHR: adjusted hazard ratio
APH: adolescent living with perinatally acquired HIV
ART: antiretroviral therapy
BIPAI: Baylor International Pediatric AIDS Initiative at Texas Children’s Hospital
CCASAnet: Caribbean, Central and South America Network for HIV Research
CI: confidence interval
CIG: country income group
CIPHER: Collaborative Initiative for Paediatric HIV Education and Research
EPPICC: European Pregnancy and Paediatric HIV Cohort Collaboration
HAZ: height-for-age Z-score; HR, hazard ratio
IeDEA: International Epidemiology Databases to Evaluate AIDS
IPW: inverse probability weighting
IQR: interquartile range
LTFU: lost to follow-up
MI: multiple imputation
MSF: Médecins Sans Frontières
PHACS AMP: Pediatric HIV/AIDS Cohort Study Adolescent Master Protocol
UCT HREC: University of Cape Town Health Research Ethics Committee
uHR: unadjusted hazard ratio
WHO: World Health Organization
