# Supplementary material for: The epidemiology of adolescents living with perinatally acquired HIV: A cross-region global cohort analysis
Source: PLoS Med. 2018 Mar 1;15(3):e1002514. doi: 10.1371/journal.pmed.1002514 (PMC5832192; doi:10.1371/journal.pmed.1002514)
Supplement: S1 Text — (DOCX) [file pmed.1002514.s002.docx]

**S2 Text**

**The Collaborative Initiative for Paediatric HIV Education and Research (CIPHER) Global Cohort Collaboration**

**CIPHER Adolescent Project Team:** Amy L. Slogrove, University of Cape Town, South Africa (data curation, formal analysis, methodology, visualization, writing – original draft preparation); Michael Schomaker, University of Cape Town, South Africa (conceptualization, data curation, formal analysis, methodology, resources, software, supervision, validation, visualization, writing – review & editing); Mary-Ann Davies, University of Cape Town, South Africa (conceptualization, data curation, project administration, supervision, writing – review & editing); Paige Williams, Harvard T. H. Chan School of Public Health, USA (conceptualization, supervision, writing – review & editing); Suna Balkan, Médecins Sans Frontières, France (conceptualization, supervision, writing – review & editing); Jihane Ben-Farhat, Epicentre, Médecins Sans Frontières, France (conceptualization, supervision, writing – review & editing); Nancy Calles, Baylor International Pediatric AIDS Initiative *at Texas Children’s Hospital*-USA, USA (conceptualization, writing – review & editing); Kulkanya Chokephaibulkit, Siriraj Hospital, Mahidol University, Thailand (investigation, writing – review & editing); Charlotte Duff, MRC Clinical Trials Unit at University College London, London, UK (conceptualization, data curation, writing – review & editing); Tonah François Eboua, CHU Yopougon, Côte d'Ivoire (conceptualization, writing – review & editing); Adeodata Kekitiinwa, Baylor International Pediatric AIDS Initiative *at Texas Children’s Hospital* -Uganda, Uganda (conceptualization, writing – review & editing); Nicky Maxwell, University of Cape Town, South Africa (conceptualization, data curation, writing – review & editing); Jorge Pinto, School of Medicine, Federal University of Minas Gerais, Brazil (conceptualization, writing – review & editing); George Seage III, Harvard T. H. Chan School of Public Health, USA (conceptualization, project administration, supervision, writing – review & editing); Chloe Teasdale, ICAP-Columbia University, Mailman School of Public Health, USA (conceptualization, data curation, writing – review & editing); Sebastian Wanless, Baylor International Pediatric AIDS Initiative *at Texas Children’s Hospital*-USA, USA (conceptualization, data curation, writing – review & editing); Josiane Warszawski, French Institute of Health and Medical Research, France (conceptualization, writing – review & editing); Kara Wools-Kaloustian, Indiana University School of Medicine, USA (conceptualization, supervision, writing – review & editing); Marcel Yotebieng, College of Public Health, Ohio State University, USA (conceptualization, writing – review & editing); Ali Judd*, MRC Clinical Trials Unit at University College London, London, UK (project co-chair, conceptualization, project administration, supervision, writing – review & editing); Valériane Leroy*, Inserm, U1027, Université Toulouse 3, France (project co-chair, conceptualization, supervision, writing – review & editing);

***** Contributed equally as project co-chairs.

**Project Oversight Group:** CIPHER Cohort Collaboration Data Centre at Centre for Infectious Disease Epidemiology and Research, University of Cape Town, South Africa - Mary-Ann Davies, (conceptualization, data curation, project administration, supervision, writing – review & editing); Nicky Maxwell (conceptualization, data curation, writing – review & editing); Michael Schomaker (conceptualization, formal analysis, supervision, writing – review & editing); Venessa Timmerman, (data curation, writing – review & editing); CIPHER Post-doctoral grantee – Amy L. Slogrove, Centre for Infectious Disease Epidemiology and Research University of Cape Town, South Africa (data curation, formal analysis, writing – original draft preparation); EPPICC – Jeannie Collins, MRC Clinical Trials Unit at University College London, London, UK (conceptualization, supervision, writing – review & editing); Charlotte Duff, MRC Clinical Trials Unit at University College London, London, UK (data curation, writing – review & editing); Ruth Goodall, MRC Clinical Trials Unit at University College London, London, UK (conceptualization, supervision, writing – review & editing); Ali Judd, MRC Clinical Trials Unit at University College London, London, UK (project co-chair, conceptualization, project administration, supervision, writing – review & editing); Colette Smith, MRC Clinical Trials Unit at University College London, London, UK (conceptualization, supervision, writing – review & editing); IeDEA East Africa - Kara Wools-Kaloustian, Indiana University School of Medicine, USA (conceptualization, investigation, supervision, writing – review & editing); IeDEA West Africa – Valériane Leroy, Inserm, U1027, Université Toulouse 3, France (Project Co-Chair, conceptualization, supervision, writing – review & editing); PHACS/IMPAACT - Kunjal Patel, Harvard T. H. Chan School of Public Health, USA (conceptualization, supervision, writing – review & editing); George Seage III, Harvard School of Public Health, USA (conceptualization, project administration, supervision, writing – review & editing); Paige Williams, Harvard T. H. Chan School of Public Health, USA (conceptualization, supervision, writing – review & editing).

**CIPHER Cohort Collaboration Steering Committee:** BIPAI - Mary Paul, Baylor International Pediatric AIDS Initiative *at Texas Children’s Hospital*, USA (conceptualization, supervision, writing – review & editing); EPPICC - Diana Gibb, MRC Clinical Trials Unit at University College London, London, UK (conceptualization, supervision, writing – review & editing); Ali Judd, MRC Clinical Trials Unit at University College London, London, UK (project co-chair, conceptualization, project administration, supervision, writing – review & editing); IeDEA Southern Africa - Mary-Ann Davies, University of Cape Town, South Africa (conceptualization, data curation, project administration, supervision, writing – review & editing); IeDEA-East Africa - Rachel Vreeman, Indiana University (conceptualization, supervision, writing – review & editing); Médecins Sans Frontières - Suna Balkan, MSF Medical Department, France (conceptualization, supervision, writing – review & editing); Jihane Ben-Farhat, Epicentre, MSF, France (conceptualization, supervision, writing – review & editing); Optimal Models (ICAP) - Elaine Abrams, ICAP-Columbia University, Mailman School of Public Health, USA (conceptualization, supervision, writing – review & editing); PHACS/IMPAACT - Rohan Hazra, US National Institutes of Health, NICHD, USA (conceptualization, supervision, writing – review & editing); George Seage III, Harvard T. H. Chan School of Public Health, USA (conceptualization, project administration, supervision, writing – review & editing); Russell Van Dyke, Tulane University, USA (supervision, writing – review & editing).

**CIPHER Executive Committee:** Linda-Gail Bekker, Desmond Tutu HIV Centre, University of Cape Town, South Africa (conceptualization, funding acquisition, supervision, writing – review & editing); Lynne Mofenson, Elizabeth Glaser Pediatric AIDS Foundation, USA (conceptualization, supervision, writing – review & editing); Marissa Vicari, International AIDS Society, Switzerland (conceptualization, funding acquisition, project administration, supervision, writing – review & editing); Shaffiq Essajee, World Health Organization, Switzerland (conceptualization, supervision, writing – review & editing); Martina Penazzato, World Health Organization, Switzerland (conceptualization, supervision, writing – review & editing).

**Representatives of contributing networks:**

**Baylor International Pediatric AIDS Initiative *at Texas Children’s* Hospital**: Botswana, Gabriel Anabwani (investigation, writing – review & editing); Lesotho, Edith Q. Mohapi (investigation, writing – review & editing); Malawi, Peter N. Kazembe (investigation, writing – review & editing); Swaziland, Makhosazana Hlatshwayo (investigation, writing – review & editing); Tanzania, Mwita Lumumba (investigation, writing – review & editing); Uganda, Adeodata Kekitiinwa-Rukyalekere (investigation, writing – review & editing; Data Manager - Sebastien Wanless (conceptualization, data curation, writing – review & editing).

**EPPICC:** Belgium, Hospital St Pierre Cohort, Tessa Goetghebuer, Hospital St Pierre, Brussels (investigation, writing – review & editing); Europe, European Collaborative Study, Claire Thorne, UCL Great Ormond Street Institute of Child Health, University College London, UK (investigation, writing – review & editing); France, French Perinatal Cohort Study, Josiane Warszawski, INSERM, France (investigation, writing – review & editing); Italy, Italian Register for HIV Infection in Children, Luisa Galli, Università degli Studi di Firenze (investigation, writing – review & editing); Netherlands, ATHENA, Annemarie van Rossum, Erasmus MC University Medical Center Rotterdam-Sophia Children’s Hospital (investigation, writing – review & editing); PENTA Foundation, Carlo Giaquinto (investigation, writing – review & editing); Poland, Polish Paediatric Cohort, Magdalena Marczynska, Medical University of Warsaw, Hospital of Infectious Diseases in Warsaw (investigation, writing – review & editing); Portugal Centro Hospitalar do Porto, Laura Marques, Centro Hospitalar do Porto, Porto (investigation, writing – review & editing), Lisbon Cohort, Filipa Prata, Hospital de Santa Maria, Lisbon, Portugal (investigation, writing – review & editing); Romania, Victor Babes Hospital Cohort, Luminita Ene, Victor Babes Hospita, Bucharest (investigation, writing – review & editing); Russian Federation, Republican Hospital of Infectious Diseases, Liubov Okhonskaia, Republican Hospital of Infectious Diseases, St Petersburg (investigation, writing – review & editing); Spain, CoRISPE-1, Pablo Rojo, Hospital Doce de Octubre, Madrid (investigation, writing – review & editing), CoRISPE—cat, Claudia Fortuny, Hospital Sant Joan de Déu, Universitat de Barcelona, Barcelona (investigation, writing – review & editing); Sweden, Karolinska University Hospital, Lars Naver, Karolinska University Hospital (investigation, writing – review & editing); Switzerland, Swiss Mother and Child HIV Cohort Study, Christoph Rudin, University Children’s Hospital, Basel (investigation, writing – review & editing); Thailand, Programs for HIV Prevention and Treatment (PHPT) Thailand, Sophie Le Coeur, Faculty of Associated Medical Sciences, Chiang Mai University and the ﻿Institut de recherche pour le développement﻿, France (investigation, writing – review & editing); UK and Ireland, CHIPS UK and Ireland, Ali Judd MRC Clinical Trials Unit at University College London, London, UK (Project Co-Chair, conceptualization, supervision, writing – review & editing), NHSPC UK and Ireland, Claire Thorne, UCL Great Ormond Street Institute of Child Health, University College London, UK (investigation, writing – review & editing); Ukraine, Ukraine Paediatric HIV Cohort Study, Alla Volokha, Shupyk National Medical Academy of Postgraduate Education, Kiev (investigation, writing – review & editing).

**CCASAnet:** Jorge Pinto, Department of Pediatrics, School of Medicine, Federal University of Minas Gerais, Brazil (conceptualization, investigation, writing – review & editing); Vanessa Rouzier, GHESKIO Center, Port-au-Prince, Haiti (investigation, writing – review & editing); Regina Succi, Universidade Federal de São Paulo, Brazil (investigation, writing – review & editing).

**IeDEA** **Asia-Pacific:** Kulkanya Chokephaibulkit, Siriraj Hospital, Mahidol University, Bangkok, Thailand (investigation, writing – review & editing); Annette Sohn, Treat Asia/amfAR, Bangkok, Thailand (conceptualization, investigation, supervision, writing – review & editing); Azar Kariminia, Kirby Institute, University of New South Wales, Sydney, Australia (data curation, writing – review & editing).

**IeDEA Central Africa:** Marcel Yotebieng, College of Public Health, Ohio State University, Columbus, USA (conceptualization, investigation, writing – review & editing); Andrew Edmonds, The Gillings School of Public Health, University of North Carolina at Chapel Hill, USA (investigation, writing – review & editing); Patricia Lelo, Pediatric Hospital Kalembe Lembe, Lingwala, Kinshasa, Demogratic Republic of Congo (investigation, writing – review & editing).

**IeDEA East Africa**: Samuel Ayaya, Academic Model Providing Access to Healthcare (AMPATH), Eldoret, Kenya (investigation, writing – review & editing); Patricia Ongwen, Family AIDS Care and Education Services, Kenya Medical Resarch Institute, Kisumu, Kenya (investigation, writing – review & editing); Rachel Vreeman, Indiana University School of Medicine, Department of Pediatrics, IU Center for Global Health, Indianapolis, Indiana (conceptualization, investigation, supervision, writing – review & editing); Kara Wools-Kaloustian, Indiana University School of Medicine, Department of Medicine, Division of Infectious Diseases, Indianapolis, Indiana (conceptualization, investigation, supervision, writing – review & editing).

**IeDEA Southern Africa:** Laura F. Jefferys, SolidarMed Lesotho, Mozambique and Zimbabwe (investigation, writing – review & editing); Sam Phiri, Lighthouse Trust Clinic, Malawi (investigation, writing – review & editing); Mwangelwa Mubiana-Mbewe, Center for Infectious Disease Research in Zambia, Zambia (investigation, writing – review & editing); Shobna Sawry, Wits Reproductive Health and HIV Institute, Faculty of Health Sciences, University of the Witwatersrand and Harriet Shezi Children’s Clinic, Chris Hani Baragwanath Hospital, South Africa (investigation, writing – review & editing).

**IeDEA West** **Africa:** Tanoh Francois Eboua, Yopougon University Hospital, University Félix Houphouët-Boigny, Abidjan, Ivory Coast (investigation, writing – review & editing); Lorna Renner, Korle Bu Teaching Hospital, Accra, Ghana (investigation, writing – review & editing); Mariam Sylla, CHU Gabriel Touré, Bamako, Mali (investigation, writing – review & editing).

**IMPAACT:** Mark J. Abzug, University of Colorado School of Medicine and Children’s Hospital Colorado, USA (investigation, writing – review & editing); Paige Williams, Harvard T. H. Chan School of Public Health, USA (conceptualization, supervision, writing – review & editing); Myron Levin, University of Colorado, USA (investigation, writing – review & editing); James Oleske, Rutgers - New Jersey Medical School, USA (investigation, writing – review & editing); Miriam Chernoff, Harvard T. H. Chan School of Public Health, USA (investigation, writing – review & editing); Rohan Hazra, US National Institutes of Health, NICHD, USA (supervision, writing – review & editing); Shirley Traite, Harvard T. H. Chan School of Public Health, USA(data curation, writing – review & editing); Murli Purswani, Bronx-Lebanon Hospital Center, USA (investigation, writing – review & editing); George Seage III, Harvard T. H. Chan School of Public Health, USA (conceptualization, supervision, writing – review & editing); Russell Van Dyke, Tulane University, USA (supervision, writing – review & editing).

**Médecins Sans Frontières:** Suna Balkan, MSF, France (conceptualization, supervision, writing – review & editing); Jihane Ben-Farhat, Epicentre, MSF, France (conceptualization, supervision, writing – review & editing).

**Optimal Models/ICAP:** Elaine Abrams, ICAP-Columbia University, Mailman School of Public Health, USA (conceptualization, supervision, writing – review & editing); Chloe Teasdale, ICAP-Columbia University, Mailman School of Public Health, USA, (conceptualization, supervision, writing – review & editing

**PHACS:** Russell Van Dyke, Tulane University, USA (supervision, writing – review & editing); George Seage III, Harvard T. H. Chan School of Public Health, USA (conceptualization, supervision, writing – review & editing); Rohan Hazra, US National Institutes of Health, NICHD, USA (supervision, writing – review & editing); Ellen Chadwick Feinberg School of Medicine, Northwestern University, USA (investigation, writing – review & editing); Paige Williams, Harvard T. H. Chan School of Public Health, USA (conceptualization, supervision, writing – review & editing); Kunjal Patel. Harvard T. H. Chan School of Public Health, USA (supervision, writing – review & editing).
