# Supplementary material for: The epidemiology of adolescents living with perinatally acquired HIV: A cross-region global cohort analysis
Source: PLoS Med. 2018 Mar 1;15(3):e1002514. doi: 10.1371/journal.pmed.1002514 (PMC5832192; doi:10.1371/journal.pmed.1002514)
Supplement: S2 Text — (DOCX) [file pmed.1002514.s003.docx]

**S1 Text**

**Acknowledgements**

The following sites, their personnel and patients are acknowledged for their contribution to the participating networks that made the CIPHER Cohort Collaboration Adolescent Project possible:

**BIPAI**: Baylor Botswana, Gabriel Anabwani; Baylor Lesotho, Edith Mohapi; Baylor Malawi, Peter N. Kazembe; Baylor Swaziland, Makhosazana Hlatshwayo; Baylor Tanzania, Mwita Lumumba; Baylor Uganda, Adeodata Kekitiinwa-Rukyalekere. Baylor International Pediatric AIDS Initiative *at Texas Chidlren’s Hospital* Founder, Mark Kline.

**EPPICC:** *Belgium* - Hospital St Pierre Cohort, Brussels: Tessa Goetghebuer, MD, PhD; Marc Hainaut, MD PhD; Evelyne Van der Kelen, research nurse; Marc Delforge, data manager. *France* - French Perinatal Cohort Study/Enquête Périnatale Française, ANRS EPF-CO10. Coordinating center, INSERM U1018, team 4: Josiane Warszawski, Jerome Le Chenadec, Elisa Ramos, Olivia Dialla, Thierry Wack, Corine Laurent, Lamya Ait si Selmi, Isabelle Leymarie, Fazia Ait Benali, Maud Brossard, Leila Boufassa. Hôpital Louis Mourier, Colombes, Dr Corinne Floch-Tudal; Groupe Hospitalier Cochin Tarnier Port-Royal, Paris, Dr Ghislaine Firtion; Centre Hospitalier Intercommunal, Creteil, Dr Isabelle Hau; Centre Hospitalier Général, Villeneuve Saint Georges, Dr Anne Chace; Centre Hospitalier Général- Hôpital Delafontaine, Saint-Denis, Dr Pascal Bolot; Groupe Hospitalier Necker, Paris, Pr Stéphane Blanche; Centre hospitalier Francilien Sud, Corbeil Essonne, Dr Michèle Granier; Hôpital Antoine Béclère, Clamart, Pr Philippe Labrune; Hôpital Jean Verdier, Bondy, Dr Eric Lachassine; Hôpital Trousseau, Paris, Dr Catherine Dollfus **;** Hôpital Robert Debré, Paris, Dr Martine Levine; Hôpital Bicêtre, Le Kremlin Bicëtre, Dr Corinne Fourcade; Centre Hospitalier Intercommunal, Montreuil, Dr Brigitte Heller- Roussin; Centre Hospitalier Pellegrin, Bordeaux, Dr Camille Runel-Belliard; CHU Paule de Viguier, Toulouse, Dr Joëlle Tricoire; CHU Hôpital de l'Archet II, Nice, Dr Fabrice Monpoux; Groupe Hospitalier de la Timone, Marseille; CHU Hôpital Jean Minjoz, Besancon, Dr Catherine Chirouze; CHU Nantes Hotel Dieu, Nantes, Dr Véronique Reliquet; CHU Caen, Caen, Pr Jacques Brouard; Institut d’Hématologie et Oncologie Pédiatrique, Lyon, Dr Kamila Kebaili; CHU Angers, Angers, Dr Pascale Fialaire; CHR Arnaud de Villeneuve, Montpellier, Dr Muriel Lalande; CHR Jeanne de Flandres, Lille, Dr Françoise Mazingue; Hôpital Civil, Strasbourg, Dr Maria Luisa Partisani. *Germany* - German Paediatric & Adolescent HIV Cohort (GEPIC): Dr Christoph Königs, Dr Stephan Schultze-Strasser. German clinical centers: Hannover Medical School, Dr. U. Baumann; Pediatric Hospital Krefeld, Dr. T. Niehues; University Hospital Düsseldorf, Dr. J. Neubert; University Hospital Hamburg, Dr. R. Kobbe; Charite Berlin, Dr. C. Feiterna-Sperling; University Hospital Frankfurt, Dr. C. Königs; University Hospital Mannheim, Dr. B. Buchholz; Munich University Hospital, Dr. G. Notheis. *Greece* - Greek cohort: Vana Spoulou. *Italy* - Italian Register for HIV infection in Children. Coordinators: Maurizio de Martino (Florence), Pier Angelo Tovo (Turin). Participants: Osimani Patrizia (Ancona), Domenico Larovere (Bari), Maurizio Ruggeri (Bergamo), Giacomo Faldella, Francesco Baldi (Bologna) Raffaele Badolato (Brescia), Carlotta Montagnani, Elisabetta Venturini, Catiuscia Lisi (Florence), Antonio Di Biagio, Lucia Taramasso (Genua), Vania Giacomet, Paola Erba, Susanna Esposito, Rita Lipreri, Filippo Salvini, Claudia Tagliabue (Milan), Monica Cellini (Modena), Eugenia Bruzzese, Andrea Lo Vecchio (Naples), Osvalda Rampon, Daniele Donà (Padua), Amelia Romano (Palermo), Icilio Dodi (Parma), Anna Maccabruni (Pavia), Rita Consolini (Pisa), Stefania Bernardi, Hyppolite Tchidjou Kuekou, Orazio Genovese (Rome), Paolina Olmeo (Sassari), Letizia Cristiano (Taranto), Antonio Mazza (Trento), Clara Gabiano, Silvia Garazzino (Turin), Antonio Pellegatta (Varese). *Netherlands* - The ATHENA database is maintained by Stichting HIV Monitoring through the Centre for Infectious Disease Control of the National Institute for Public Health and the Environment. The following clinical centres contributed: Emma Kinderziekenhuis, Academic Medical Centre of the University of Amsterdam: HIV treating physicians: D. Pajkrt, H.J. Scherpbier. HIV nurse consultants: A.M. Weijsenfeld, A. van der Plas. HIV clinical virologists/chemists: S. Jurriaans, N.K.T. Back, H.L. Zaaijer, B. Berkhout, M.T.E. Cornelissen, C.J. Schinkel, K.C. Wolthers. Erasmus MC–Sophia, Rotterdam: HIV treating physicians: P.L.A. Fraaij, A.M.C. van Rossum. HIV nurse consultants: L.C. van der Knaap, E.G. Visser. HIV clinical virologists/chemists: C.A.B. Boucher, M.P.G Koopmans, J.J.A van Kampen, S.D. Pas. Radboudumc, Nijmegen: HIV treating physicians: S.S.V. Henriet, M. van de Flier, K. van Aerde. HIV nurse consultants: R. Strik-Albers. HIV clinical virologists/chemists: J. Rahamat-Langendoen, F.F. Stelma. Universitair Medisch Centrum Groningen, Groningen: HIV treating physicians: E. H. Schölvinck. HIV nurse consultants: H. de Groot-de Jonge. HIV clinical virologists/chemists: H.G.M. Niesters, C.C. van Leer-Buter, M. Knoester. Wilhelmina Kinderziekenhuis, UMCU, Utrecht: HIV treating physicians: L.J. Bont, S.P.M. Geelen, T.F.W. Wolfs. HIV nurse consultants: N. Nauta. HIV clinical virologists/chemists: C.W. Ang, R. van Houdt, A.M. Pettersson, C.M.J.E. Vandenbroucke-Grauls. The Co-ordinating Centre is acknowledged. Director: P. Reiss. Data analysis: D.O. Bezemer, A.I. van Sighem, C. Smit, F.W.M.N. Wit, T.S. Boender. Data management and quality control: S. Zaheri, M. Hillebregt, A. de Jong. Data monitoring: D. Bergsma, S. Grivell, A. Jansen, M. Raethke, R. Meijering. Data collection: L. de Groot, M. van den Akker, Y. Bakker, E. Claessen, A. El Berkaoui, J. Koops, E. Kruijne, C. Lodewijk, L. Munjishvili, B. Peeck, C. Ree, R. Regtop, Y. Ruijs, T. Rutkens, M. Schoorl, A. Timmerman, E. Tuijn, L. Veenenberg, S. van der Vliet, A. Wisse, T. Woudstra. Patient registration: B. Tuk. *Poland* - Polish paediatric cohort: Head of the team: Prof Magdalena Marczyńska, MD, PhD. Members of the team: Jolanta Popielska, MD, PhD; Maria Pokorska-Śpiewak, MD, PhD; Agnieszka Ołdakowska, MD, PhD; Konrad Zawadka, MD, PhD; Urszula Coupland, MD, PhD. Administration assistant: Małgorzata Doroba. Affiliation: Medical University of Warsaw, Poland, Department of Children’s Infectious Diseases; Hospital of Infectious Diseases in Warsaw, Poland. *Portugal -*  Centro Hospitalar do Porto: Laura Marques, Carla Teixeira, Alexandre Fernandes. Portugal: Hospital de Santa Maria/CHLN: Filipa Prata. *Romania* - "Victor Babes" Hospital Cohort, Bucharest: Dr Luminita Ene. *Russia* - Federal State-owned Institution "Republican Clinical Infectious Diseases Hospital" of the Ministry of Health of the Russian Federation, St Petersburg: Liubov Okhonskaia, Evgeny Voronin, Milana Miloenko, Svetlana Labutina. *Spain* - CoRISPE-cat, Catalonia: Hospital Universitari Vall d’Hebron, Barcelona (Pere Soler-Palacín, Maria Antoinette Frick and Santiago Pérez-Hoyos (statistician)), Hospital Universitari del Mar, Barcelona (Antonio Mur, Núria López), Hospital Universitari Germans Trias i Pujol, Badalona (María Méndez), Hospital Universitari JosepTrueta, Girona (Lluís Mayol), Hospital Universitari Arnau de Vilanova, Lleida (Teresa Vallmanya), Hospital Universitari Joan XXIII, Tarragona (Olga Calavia), Consorci Sanitari del Maresme, Mataró (Lourdes García), Hospital General de Granollers (Maite Coll), Corporació Sanitària Parc Taulí, Sabadell (Valentí Pineda), Hospital Universitari Sant Joan, Reus (Neus Rius), Fundació Althaia, Manresa (Núria Rovira), Hospital Son Espases, Mallorca (Joaquín Dueñas) and Hospital Sant Joan de Déu, Esplugues (Clàudia Fortuny, Antoni Noguera-Julian). CoRISPE-S and Madrid cohort: María José Mellado, Luis Escosa, Milagros García Hortelano, Talía Sainz (Hospital La Paz);María Isabel González- Tomé, Pablo Rojo, Daniel Blázquez (Hospital Doce de Octubre, Madrid); José Tomás Ramos (Hospital Clínico San Carlos, Madrid); Luis Prieto, Sara Guillén (Hospital de Getafe); María Luisa Navarro, Jesús Saavedra, Mar Santos, Mª Angeles Muñoz, Beatriz Ruiz, Carolina Fernandez Mc Phee, Santiago Jimenez de Ory,Susana Alvarez (Hospital Gregorio Marañón); Miguel Ángel Roa (Hospital de Móstoles); José Beceiro (Hospital Príncipe de Asturias, Alcalá de Henares); Jorge Martínez (Hospital Niño Jesús, Madrid); Katie Badillo (Hospital de Torrejón); Miren Apilanez (Hospital de Donostia, San Sebastián); Itziar Pocheville (Hospital de Cruces, Bilbao); Elisa Garrote (Hospital de Basurto, Bilbao); Elena Colino (Hospital Insular Materno Infantil, Las Palmas de Gran Canaria); Jorge Gómez Sirvent (Hospital Virgen de la Candelaria, Santa Cruz de Tenerife); Mónica Garzón, Vicente Román (Hospital de Lanzarote); Abián Montesdeoca, Mercedes Mateo (Complejo Universitario de Canarias, La Laguna-Tenerife),María José Muñoz, Raquel Angulo (Hospital de Poniente, El Ejido); Olaf Neth, Lola Falcón (Hospital Virgen del Rocio, Sevilla); Pedro Terol (Hospital Virgen de la Macarena, Sevilla); Juan Luis Santos (Hospital Virgen de las Nieves, Granada); David Moreno (Hospital Carlos Haya, Málaga); Francisco Lendínez (Hospital de Torrecárdenas, Almería); Ana Grande (Complejo Hospitalario Universitario Infanta Cristina, Badajoz); Francisco José Romero (Complejo Hospitalario de Cáceres); Carlos Pérez (Hospital de Cabueñes, Gijón); Miguel Lillo (Hospital de Albacete); Begoña Losada (Hospital Virgen de la Salud, Toledo); Mercedes Herranz (Hospital Virgen del Camino, Pamplona); Matilde Bustillo, Carmelo Guerrero (Hospital Miguel Servet, Zaragoza); Pilar Collado (Hospital Clínico Lozano Blesa, Zaragoza); José Antonio Couceiro (Complejo Hospitalario de Pontevedra); Amparo Pérez, Ana Isabel Piqueras, Rafael Bretón, Inmaculada Segarra (Hospital La Fe, Valencia); César Gavilán (Hospital San Juan de Alicante); Enrique Jareño (Hospital Clínico de Valencia); Elena Montesinos (Hospital General de Valencia); Marta Dapena (Hospital de Castellón); Cristina Álvarez (Hospital Marqués de Valdecilla, Santander); Ana Gloria Andrés (Hospital de León); Víctor Marugán, Carlos Ochoa (Hospital de Zamora); Santiago Alfayate, Ana Isabel Menasalvas (Hospital Virgen de la Arrixaca, Murcia); Elisa de Miguel (Complejo Hospitalario San Millán-San Pedro, Logroño) and Paediatric HIV-BioBank integrated in the Spanish AIDS Research Network and collaborating Centers. *Sweden* - Karolinska University Hospital, Stockholm (Lars Naver, Sandra Soeria-Atmadja, Vendela Hagås). *Switzerland* - Members of the Swiss HIV Cohort Study (SHCS) and the Swiss Mother and Child HIV Cohort Study: Aebi-Popp K, Asner S, Aubert V, Battegay M, Baumann M, Bernasconi E, Böni J, Brazzola P, Bucher HC, Calmy A, Cavassini M, Ciuffi A, Duppenthaler A, Dollenmaier G, Egger M, Elzi L, Fehr J, Fellay J, Francini K, Furrer H, Fux CA, Grawe C, Günthard HF (President of the SHCS), Haerry D (deputy of "Positive Council"), Hasse B, Hirsch HH, Hoffmann M, Hösli I, Kahlert C, Kaiser L, Keiser O, Klimkait T, Kovari H, Kouyos RD, Ledergerber B, Martinetti G, Martinez de Tejada B, Metzner KJ, Müller, Nicca D, Paioni P, Pantaleo G, Polli Ch, Posfay-Barbe K, Rauch A, Rudin C (Chairman of the Mother & Child Substudy), Schmid P, Scherrer AU (Head of Data Centre), Speck R, Tarr P, Thanh Lecompte M, Trkola A, Vernazza P, Wagner N, Wandeler G, Weber R, Wyler CA, Yerly S. *Thailand* - Program for HIV Prevention & Treatment (PHPT). Participating hospitals: Lamphun: Pornpun Wannarit; Phayao Provincial Hospital: Pornchai Techakunakorn; Chiangrai Prachanukroh: Rawiwan Hansudewechakul; Chiang Kham: Vanichaya Wanchaitanawong; Phan: Sookchai Theansavettrakul; Mae Sai: Sirisak Nanta; Prapokklao: Chaiwat Ngampiyaskul; Banglamung: Siriluk Phanomcheong; Chonburi: Suchat Hongsiriwon; Rayong: Warit Karnchanamayul; Bhuddasothorn Chacheongsao: Ratchanee Kwanchaipanich; Nakornping: Suparat Kanjanavanit; Somdej Prapinklao: Nareerat Kamonpakorn, Maneeratn Nantarukchaikul; Bhumibol Adulyadej: Prapaisri Layangool, Jutarat Mekmullica; Pranangklao: Paiboon Lucksanapisitkul, Sudarat Watanayothin; Buddhachinaraj: Narong Lertpienthum; Hat Yai: Boonyarat Warachit; Regional Health Promotion Center 6, Khon Kaen: Sansanee Hanpinitsak; Nong Khai: Sathit Potchalongsin; Samutsakhon: Pimpraphai Thanasiri, Sawitree Krikajornkitti; Phaholpolphayuhasena: Pornsawan Attavinijtrakarn; Kalasin: Sakulrat Srirojana; Nakhonpathom: Suthunya Bunjongpak; Samutprakarn: Achara Puangsombat; Mahasarakam: Sathaporn Na-Rajsima; Roi-et: Pornchai Ananpatharachai; Sanpatong: Noppadon Akarathum; Vachira Phuket: Weerasak Lawtongkum; Chiangdao: Prapawan Kheunjan, Thitiporn Suriyaboon, Airada Saipanya. Data management team: Kanchana Than-in-at, Nirattiya Jaisieng, Rapeepan Suaysod, Sanuphong Chailoet, Naritsara Naratee, and Suttipong Kawilapat. *Ukraine* - Paediatric HIV Cohort: Dr T. Kaleeva, Dr Y. Baryshnikova (Odessa Regional Centre for HIV/AIDS, Dr S. Soloha (Donetsk Regional Centre for HIV/AIDS), Dr N. Bashkatova (Mariupol AIDS Center), Dr I. Raus (Kiev City Centre for HIV/AIDS), Dr O. Glutshenko, Dr Z. Ruban (Mykolaiv Regional Centre for HIV/AIDS), Dr N. Prymak (Kryvyi Rih), Dr G. Kiseleva (Simferopol), Dr H. Bailey (UCL, London, UK). *UK & Ireland* - Collaborative HIV Paediatric Study (CHIPS): CHIPS Steering Committee: Hermione Lyall, Karina Butler, Katja Doerholt, Caroline Foster, Nigel Klein, Esse Menson, Andrew Riordan, Delane Shingadia, Gareth Tudor-Williams, Pat Tookey, Steve Welch. MRC Clinical Trials Unit: Intira Jeannie Collins, Claire Cook, Donna Dobson, Keith Fairbrother, Diana M. Gibb, Ali Judd, Lynda Harper, Francesca Parrott, Anna Tostevin, Nadine Van Looy. Participating hospitals: Republic of Ireland: Our Lady's Children’s Hospital Crumlin, Dublin: K Butler, A Walsh. UK: Birmingham Heartlands Hospital, Birmingham: S Scott, Y Vaughan, S Welch; Blackpool Victoria Hospital, Blackpool: N Laycock; Bristol Royal Hospital for Children, Bristol: J Bernatoniene, A Finn, L Hutchison; Calderdale Royal Hospital, Halifax: G Sharpe; Central Middlesex Hospital, London: A Williams; Chelsea and Westminster Hospital, London: EGH Lyall, P Seery; Coventry & Warwickshire University Hospital, Coventry: P Lewis, K Miles; Derbyshire Children’s Hospital, Derby: B Subramaniam; Derriford Hospital, Plymouth: L Hutchinson, P Ward; Ealing Hospital, Middlesex: K Sloper; Eastbourne District General Hospital, Eastbourne: G Gopal; Glasgow Royal Hospital for Sick Children, Glasgow: C Doherty, R Hague, V Price; Great Ormond St Hospital for Children, London: A Bamford, H Bundy, M Clapson, J Flynn, DM Gibb, N Klein, V Novelli, D Shingadia; Halliwell Children’s Centre, Bolton: P Ainsley-Walker; Harrogate District Hospital, Harrogate: P Tovey; Homerton University Hospital, London: D Gurtin; Huddersfield Royal Infirmary, Huddersfield: JP Garside; James Cook Hospital, Middlesbrough: A Fall; John Radcliffe Hospital, Oxford: D Porter, S Segal; King's College Hospital, London: C Ball, S Hawkins; Leeds General Infirmary, Leeds: P Chetcuti, M Dowie; Leicester Royal Infirmary, Leicester: S Bandi, A McCabe; Luton and Dunstable Hospital, Luton: M Eisenhut; Mayday University Hospital, Croydon: J Handforth; Milton Keynes General Hospital, Milton Keynes: PK Roy; Newcastle General Hospital, Newcastle: T Flood, A Pickering; Newham General Hospital, London: S Liebeschuetz; Norfolk & Norwich Hospital, Norwich: C Kavanagh; North Manchester General Hospital, Manchester: C Murphy, K Rowson, T Tan; North Middlesex Hospital, London: J Daniels, Y Lees; Northampton General Hospital, Northampton: E Kerr, F Thompson; Northwick Park Hospital Middlesex; M Le Provost, A Williams; Nottingham City Hospital, Nottingham: L Cliffe, A Smyth, S Stafford; Queen Alexandra Hospital, Portsmouth: A Freeman; Raigmore Hospital, Inverness: T Reddy; Royal Alexandra Hospital, Brighton: K Fidler; Royal Belfast Hospital for Sick Children, Belfast: S Christie; Royal Berkshire Hospital, Reading: A Gordon; Royal Children’s Hospital, Aberdeen: D Rogahn; Royal Cornwall Hospital, Truro: S Harris, L Hutchinson; Royal Devon and Exeter Hospital, Exeter: A Collinson, L Hutchinson; Royal Edinburgh Hospital for Sick Children, Edinburgh: L Jones, B Offerman; Royal Free Hospital, London: V Van Someren; Royal Liverpool Children’s Hospital, Liverpool: C Benson, A Riordan; Royal London Hospital, London: A Riddell; Royal Preston Hospital, Preston: R O’Connor; Salisbury District General Hospital, Salisbury: N Brown; Sheffield Children's Hospital, Sheffield: L Ibberson, F Shackley; Southampton General Hospital, Southampton: SN Faust, J Hancock; St George's Hospital, London: K Doerholt, S Donaghy, K Prime, M Sharland, S Storey; St Luke’s Hospital, Bradford: S Gorman; St Mary’s Hospital, London: EGH Lyall, C Monrose, P Seery, G Tudor-Williams, S Walters; St Thomas’ Hospital (Evelina Children’s Hospital), London: R Cross, E Menson; Torbay Hospital, Torquay: J Broomhall, L Hutchinson; University Hospital Lewisham, London: D Scott, J Stroobant; University Hospital of North Staffordshire, Stoke On Trent: A Bridgwood, P McMaster; University Hospital of Wales, Cardiff: J Evans, T Gardiner; Wexham Park, Slough: R Jones; Whipps Cross Hospital, London: K Gardiner.

**CCASAnet:** Fundación Huésped, Argentina, Pedro Cahn; Universidade Federal de Minas Gerais, Brazil, Jorge Pinto; Universidade Federal de São Paulo, Brazil, Regina Célia de Menezes Succi; Les Centres GHESKIO, Haiti, Jean William Pape; Hospital Escuela Universitario, Honduras, Marco Tulio Luque; Instituto Hondureño de Seguridad Social, Honduras, Denis Padgett.

**IeDEA Asia-Pacific:** National Centre for HIV/AIDS, Dermatology and STDs, Phnom Penh, Cambodia, PS Ly*, and V Khol; New Hope for Cambodian Children, Phnom Penh, Cambodia, J Tucker; YRGCARE Medical Centre, CART CRS, Chennai, India, N Kumarasamy*, S Saghayam, and E Chandrasekaran; Sanglah Hospital, Udayana University, Bali, Indonesia, DK Wati*, D Vedaswari, and IY Malino; Cipto Mangunkusumo – Faculty of Medicine Universitas Indonesia, Jakarta, Indonesia, N Kurniati*, and D Muktiarti; Hospital Likas, Kota Kinabalu, Malaysia, SM Fong*, M Lim, and F Daut; Hospital Raja Perempuan Zainab II, Kelantan, Malaysia, NK Nik Yusoff*‡, and P Mohamad; Pediatric Institute, Hospital Kuala Lumpur, Kuala Lumpur, Malaysia, KA Razali*, TJ Mohamed, and MR Drawis; Penang Hospital, Penang, Malaysia, R Nallusamy*, and KC Chan; Department of Pediatrics, Faculty of Medicine, Chiang Mai University and Research Institute for Health Sciences, Chiang Mai, Thailand, T Sudjaritruk*, V Sirisanthana, L Aurpibul, and P Oberdorfer; Chiangrai Prachanukroh Hospital, Chiang Rai, Thailand, R Hansudewechakul*, S Denjanta, S Watanaporn, and A Kongphonoi; Division of Infectious Diseases, Department of Pediatrics, Faculty of Medicine, Khon Kaen University, Khon Kaen, Thailand, P Lumbiganon*†, P Kosalaraksa, P Tharnprisan, and T Udomphanit; PHPT-IRD UMI 174 (Institut de recherche pour le développement and Chiang Mai University), Chiang Mai, Thailand, G Jourdain; HIV-NAT, The Thai Red Cross AIDS Research Centre, Bangkok, Thailand, T Bunupuradah*, T Puthanakit, S Anugulruengkitt, and C Phadungphon; Department of Pediatrics, Faculty of Medicine Siriraj Hospital, Mahidol University, Bangkok, Thailand, K Chokephaibulkit*, K Lapphra, W Phongsamart, and S Sricharoenchai; Children’s Hospital 1, Ho Chi Minh City, Vietnam, KH Truong*, QT Du, and CH Nguyen; Children’s Hospital 2, Ho Chi Minh City, Vietnam, VC Do*, TM Ha, and VT An; National Hospital of Pediatrics, Hanoi, Vietnam, LV Nguyen*, DTK Khu, AN Pham, and LT Nguyen; Worldwide Orphans Foundation, Ho Chi Minh City, Vietnam, ON Le; TREAT Asia/amfAR -- The Foundation for AIDS Research, Bangkok, Thailand, AH Sohn*, JL Ross, and C Sethaputra; The Kirby Institute, UNSW Australia, Sydney, Australia, DA Cooper, MG Law*, and A Kariminia; (*Steering Committee members; † Current Steering Committee Chair; ‡ co-Chair).

**IeDEA Central Africa:** Burundi, Christelle Twizere – Association Nationale de Soutien aux Séropositifs; HIV-clinic (CPAMP-CHUK), Bujumbura University Hospital; Hôpital Prince Régent Charles; Democratic Republic of Congo, Marcel Yotebieng – Bomoi Health Center; Kalembe Lembe Pediatric Hospital; Rwanda, Jean D’amour Sinayobye - Bethsaid Hospital; Busanza Health Center; Gahanga Health Center; Gikondo Health Center; Kabuga Health Center; Kanombe Hospital/Rwanda Military Hospital; Kicukuri Health Center; Masaka Hospital; Nyarugunga Hospital; Women's Equity in Access to Care & Treatment.

**IeDEA East Africa:** Academic Model Providing Access to Healthcare, Kenya, Samuel Ayaya; National Institute for Medical Research, Family AIDS Care and Education Services, Kenya, Elizabeth Bukusi; National AIDS Control Program, Tanzania, Geoffrey Somi; Morogoro Regional Hospital, Tanzania, Rita Lyumuya; Tumbi Regional Hospital, Tanzania, Ngonyani Kapella; National Institute for Medical Research, Kisesa Clinic, Tanzania, Mark Urassa; Masaka Regional Referral Hospital, Ugana, Mark Ssali; Rakai Health Science Program, Uganda, Fred Nalugoda. IeDEA East Africa also acknowledges senior data manager Beverly Musick, Indiana University, USA.

**IeDEA Southern Africa:** Aid for AIDS, South Africa, Gary Maartens; Aurum Institute for Health Research, South Africa, Christopher J. Hoffmann; Centre for Infectious Disease Research in Zambia, Zambia, Michael Vinikoor; Centro de Investigaçao em Saude de Manhiça, Mozambique, Eusebio Maceta; Dignitas, Malawi, Monique van Lettow; Gugulethu Cohort (Desmond Tutu HIV Centre), South Africa, Robin Wood; Harriet Shezi Clinic, Chris Hani Baragwanath Hospital (Wits Paediatric HIV Clinics), South Africa, Shobna Sawry; Hlabisa (Africa Centre for Health & Population Studies), South Africa, Frank Tanser; Khayelitsha ART Programme, South Africa, Andrew Boulle; Kheth ‘Impilo, South Africa, Geoffrey Fatti; Lighthouse Truse clinic, Malawi, Sam Phiri; McCord Hospital, South Africa, Janet Giddy; Newlands Clinic, Zimbabwe, Cleophas Chimbetete; Queen Elizabeth Hospital, Malawi, Kennedy Malisita; Rahima Moosa Mother & Child Hospital (Wits Paediatric HIV Clinics), South Africa, Karl Technau; Red Cross War Memorial Children’s Hospital and School of Child & Adolescent Health, University of Cape Town, South Africa, Brian Eley; SolidarMed SMART Programme Lesotho, Lesotho, Christiane Fritz; SolidarMed SMART Programme Mozambique, Mozambique, Michael Hobbins; SolidarMed SMART Programme Zimbabwe, Zimbabwe, Kamelia Kamenova; Themba Lethu Clinic, Helen Joseph Hospital, South Africa, Matthew P. Fox; Tygerberg Academic Hospital, South Africa, Hans Prozesky.

**IeDEA West Africa:** *Executive Committee*: François Dabis (Principal Investigator, Bordeaux, France), Emmanuel Bissagnene (Co-Principal Investigator, Abidjan, Côte d’Ivoire), Elise Arrivé (Bordeaux, France), Patrick Coffie (Abidjan, Côte d’Ivoire), Didier Ekouevi (Abidjan, Côte d’Ivoire), Antoine Jaquet (Bordeaux, France), Valériane Leroy (Chair of the pediatric group, Toulouse, France). Benin, Cotonou: Sikiratou Koumakpaï, (CNHU Hubert Maga).Côte d’Ivoire, Abidjan: Marie-Sylvie N’Gbeche, Kouadio Kouakou (CIRBA); Madeleine Amorissani Folquet (CHU Cocody); Tanoh François Eboua (CHU Yopougon). Ghana, Accra: Lorna Renner (Korle Bu TH). Mali, Bamako: Fatoumata Dicko, Mariam Sylla (CH Gabriel Toure). Togo, Lomé: Elom Takassi (CHU Tokoin/Sylvanus Olympio). Senegal, Dakar: Haby Signate-Sy, Hélène Dior (CH Albert Royer).Burkina Faso, Ouagadougou: Diarra Yé, Fla Kouéta (CH Charles de Gaulle).

**IMPAACT:** The following sites participated in PACTG 219/219C: University of New Jersey Medical and Dental School*,* Boston Medical Center, Children’s Hospital LA, Long Beach Memorial Medical Center, Miller Children's Hospital, Harbor/UCLA Medical Center, Johns Hopkins Hospital & Health System, University of Maryland Medical Center, Texas Children's Hospital, Cook County Hospital*,* Children's Hospital of Columbus Ohio, University of Miami Miller School of Medicine, University of California San Francisco School of Medicine, Children's Hospital & Research Center Oakland, University of California San Diego, Duke University School of Medicine, University of North Carolina at Chapel Hill School of Medicine*,* Schneider Children’s Hospital*,* Harlem Hospital Center, New York University School of Medicine, Children's National Medical Center, University of Washington School of Medicine*,* University of Illinois College of Medicine at Chicago, Yale University School of Medicine*,* SUNY at Stony Brook School of Medicine, Howard University Hospital, LA County/University of Southern California Medical Center*,* University of Florida Health Science Center Jacksonville, North Broward Hospital District, Children's Diagnostic & Treatment Center*,* University of Rochester Medical Center, Golisano Children's Hospital, Medical College of Virginia, St. Jude Children's Research Hospital, University of Puerto Rico, U. Children’s Hospital AIDS*,* Children's Hospital of Philadelphia, St. Christopher’s Hospital for Children/Drexel University College of Medicine*,* Bronx-Lebanon Hospital Center, New York Medical College/Metropolitan Hospital Center, University of Massachusetts Memorial Children's Medical School, Baystate Medical Center, Connecticut Children's Medical Center*,* Medical College of Georgia School of Medicine, University of South Alabama College of Medicine, LSU Health Sciences Center, Tulane University Health Sciences Center, St. Josephs Hospital and Medical Center, Cooper University Hospital, Children's Hospital Boston, David Geffen School of Medicine at UCLA, Children's Hospital of Orange County, Children's Memorial Hospital University of Chicago, Mt. Sinai Hospital Medical Center Chicago, Columbia University Medical Center, Incarnation Children’s Center, Cornell University, Bellevue Hospital, San Francisco General, Phoenix Children's Hospital, Metropolitan Hospital Center New York, University of Cincinnati, SUNY Downstate Medical Center, Children's Hospital at Downstate, North Shore University Hospital, University of South Florida, Cornell University, Oregon Health & Science University, Children's Hospital of the King's Daughters, Lincoln Medical & Mental Health Center, Mt. Sinai School of Medicine, Emory University Hospital, San Juan City Hospital, UMDNJ - Robert Wood Johnson, Ramon Ruiz Arnau University Hospital, Medical University of South Carolina, SUNY Upstate Medical University, Wayne State University School of Medicine, Children's Hospital of Michigan, Children’s Hospital at Albany Medical Center, Children’s Medical Center of Dallas, University of Colorado at Denver and Health Sciences, Columbus Children’s Hospital, University of Florida College of Medicine, University of Mississippi Medical Center, Palm Beach County Health Department, Children’s Hospital LA, Vanderbilt University Medical Center, Washington University School of Medicine at St. Louis, St. Louis Children's Hospital, Children’s Hospital & Medical Center Seattle, St. Luke's-Roosevelt Hospital Center, Montefiore Medical Center/Albert Einstein College of Medicine, Children's Hospital Washington DC, Children’s Hospital of the King's Daughters, University of Alabama at Birmingham, Columbus Regional HealthCare System, The Medical Center, Sacred Heart Children’s Hospital/CMS of Florida, Bronx Municipal Hospital Center/Jacobi Medical Center.

The following sites participated in P1074: New Jersey Medical School, UCLA–Los Angeles/Brazil AIDS Consortium, Texas Children’s Hospital, Lurie Children’s Hospital of Chicago, Columbia University Medical Center, University of Miami Pediatric Perinatal HIV/AIDS, University of California San Diego, Mother-Child-Adolescent Program, Duke University Medical Center, Children’s Hospital of Boston, Boston Medical Center Pediatric HIV Program, New York University, Jacobi Medical Center Bronx, Children’s National Medical Center Washington, DC, Seattle Children’s Hospital, University of South Florida Tampa, San Juan City Hospital, SUNY Stony Brook, Children’s Hospital of Michigan, Howard University Washington DC, Harbor UCLA Medical Center, University of Southern California School of Medicine, University of Florida Health Science Center, University of Colorado Denver, South Florida Children’s Diagnostic and Treatment Center Fort Lauderdale, Strong Memorial Hospital University of Rochester Medical Center, Rush University Cook County Hospital Chicago, Children’s Hospital of Los Angeles, University of California San Francisco, Johns Hopkins University Baltimore, Miller Children’s Hospital, University of Maryland Baltimore, Tulane University New Orleans, University of Alabama Birmingham, The Children’s Hospital of Philadelphia, Bronx-Lebanon Hospital, St Jude’s Children’s Hospital, University of Puerto Rico Pediatric HIV/AIDS Research Program, Western New England Maternal Pediatric Adolescent AIDS.

**Médecins Sans Frontières:** The following sites contributed to the MSF Pediatric Cohorts : *Cameroon* – Douala Nylon, Douala Soboum; *Central African Republic* – Boguila, Carnot, Zemio; *Democratic Republic of Congo* – Bukavu Baraka, Katanga Dubie, Katanga Kilwa, Kimbi Lulenge, Kinshasa, Mweso, Shamwana; *Ethiopia* – Abdurafi; *India* – Manipur, Moreh, Mumbai; Malawi – Chiradzulu; Mozambique – Alto Mae, Chamanculo; *Myanmar* – Dawei, Insein, Kachin Bhamo, Kachin Moegaung, Kachin Myitkyina 1, Kachin Myitkyina 1, Kachin Phakant, Kachin Waimaw, Rakhine Maugdaw, Rachine Sittwe, Shan Laiza, Shan Lashio, Shan Muse, Yangon B, Yangon C, Yangon I, Yangon T, Yangon TIB, Yangon TIC; South Sudan – Leer, Nasir; Guinea – Conakry; *Uganada* – Arua.

**PHACS:** The following sites participated in PHACS AMP: Ann & Robert H. Lurie Children’s Hospital of Chicago: Ram Yogev; Baylor College of Medicine: William Shearer; Bronx Lebanon Hospital Center: Murli Purswani; Children's Diagnostic & Treatment Center: Ana Puga; Children’s Hospital, Boston: Sandra K. Burchett; Jacobi Medical Center: Andrew Wiznia; Rutgers - New Jersey Medical School: Arry Dieudonne; St. Christopher’s Hospital for Children: Janet S. Chen; St. Jude Children's Research Hospital: Katherine Knapp; San Juan Hospital/Department of Pediatrics: Midnela Acevedo-Flores; Tulane University School of Medicine: Margarita Silio; University of California, San Diego: Stephen A. Spector; University of Colorado Denver Health Sciences Center: Elizabeth McFarland; University of Miami: Gwendolyn Scott. Project coordination was provided by Harvard T.H. Chan School of Public Health: Julie Alperen and by Tulane University School of Medicine: Patrick Davis. Data management was provided by Frontier Science and Technology Research Foundation: Sue Siminski; Operational and regulatory support was provided by Westat Inc: Julie Davidson.

**Optimal Models-ICAP:** The following people and sites contributed to Optimal Models: *Ethiopia* -Mohamed Ahmed, Harari Regional Health Bureau; Zelalem Habtamu, Oromia Regional Health Bureau; Kassahun Hailegiorgis, Dire Dawa Regional Health Bureau; Zenebe Melaku, ICAP Ethiopia. Abomsa Hospital, Abosto HC, Adama Hospital, Addis Ketema Health Center, Adola, Ambo Hospital, Arategna Health Center, Assela Hospital, Bishoftu Hospital, Bisidimo Hospital, Bulle Hura Hospital, Chiro Hospital, Deder Hospital, Dire Dawa Health Center, Dodola, Fitche Hospital, Gedo Hospital, Gelemso Hospital, Gende Gerada Health Center, Gende Kore Health Center, Gindeberet Hospital, Ginir Hospital, Goro Health Center, Harar TB Hospital, Hiwot Fana Hospital, Jenila Health Center, Jijiga Health Center, Jimma Hospital, Karamara Hospital, Kuyu Hospital, Leghare Health Center, Limmu-Genet Hospital, Mariam Work Hospital, Melka-Jebdu Health Center, Metehara Hospital, Metu Karl Hospital, Misrak Arbegnoch Hospital, Negele Hospital, Robe Dida, Sabian Health Center, Shashemene Hospital, Sher Ethiopia Private Hospital, St. Luke (Wolisso) Hospital, Tulu Bolo Hospital, Wonji Hospital, Yabelo, Yimaji Private Hospital. *Kenya -* Mark Hawken, ICAP Kenya; Maureen Kamene Kimenye, PASCO Central province; Irene N. Mukui, National AIDS and STIs Control Programme. Abidha Health Center, Ahero Sub District Hospital, Akala Health Center, Aluor Mission, Athi River Health Center, Awasi Mission, Bar Agulu Dispensary, Bar Olengo, Bondo District Hospital, Boro Dispensary, Daniel Comboni Dispensary-Ndithini, Dienya Health Center, Gobei Dispensary, Hawinga Dispensary, Kali Dispensary, Kathiani Sub District Hospital, Kibwezi Health Center, Kikoko Mission Hospital, Kitui District hospital, Madiant District Hospital, Mahaya Health Center, Malanga Health Center, Manyuanda Dispensary, Masogo Subdistrict Hospital, Matangwe mission Hospital, Mtito Andei Health Center, Muhoroni SDH, Mulaha Dispensary, Naya Dispensary, Ndere Health Center, Ndori Dispensary, Ngiya Mission, Nyakach AIC Dispensary, Nyangoma Kogelo, Ongielo Health Center, Rangala Mission, Rera Health Center, Siaya District Hospital, Sigomere Health Center, Sikalame Health Center, Tawa Health Center, Tingwangi Health Center, Ukwala Health Center, Usigu Health Center, Uyawi Health Center, Wagai Dispensary, Yala Sub District Hospital. *Mozambique* - Josue Lima, ICAP Mozambique; Antonio Mussa, ICAP Mozambique; Américo Rafi Assan, Ministry of Health Mozambique. 17 de Setembro Health Center, 25 de Setembro Health Center – Nampula, Akumi Health Center – Nacala, Anchilo Health Center, Angoche HR, Coalane Health Center, Gurue Rural Hospital Ilha de Mocambique, Liupo Health Center, Lumbo Health Center, Malema Health Center, Marrere General Hospital, Meconta Health Center, Memba Health Center, Milange Health Center, Military Hospital-Maputo, Mocuba Rural Hospital, Moma Health Center, Monapo HR, Monapo Health Center, Mossuril HR, Nacala-Porto District Hospital, Nacala-Porto Health Center, Nacuxa HR, Nametil Health Center, Namialo Health Center, Namitoria Health Center, Nampula Central Hospital, Namuinho Health Center, Nicoadala Health Center, Pediatric Central Hospital – Nampula, Ribaue Rural Hospital; *Rwanda* - Vincent Mutabazi, Treatment and Research AIDS Center; Ruben Sahabo, ICAP Rwanda. Avega Clinic, Bethsaida Health Center, Bigogwe Health Center, Busasamana Health Center, Butare Hospital, Carrefour Polyclinic, Central Hospital-Kigali, Congo Nil Health Center, Gisenyi District Hospital, Gisenyi Prison, Gisovu Health Center, Kabaya District Hospital, Kabusunzu Health Center, Kayove Health Center, Kibuye District Hospital, Kicukiro Health Center, Kigali Central Prison, Kigufi Health Center, Kinunu Health Center, Kirambo Health Center, Kirinda District Hospital, Kivumu Health Center, Mugonero District Hospital, Muhima District Hospital, Muhororo District Hospital, Mukungu Health Center, Munzanga Health Center, Murunda District Hospital, Mushubati Health Center, Mwendo Health Center, Ndera Neuropsychiatric Hospital, Nyabirasi Health Center, Nyakiriba Health Center, Nyange A health Center, Nyange B Health Center, Ramba Health Center, Rambura Health Center, Rubengera Health Center, Rugarama Health Center, Rususa health Center, Shyira District Hospital; *Tanzania* - Gretchen Antelman, ICAP Tanzania; Redempta Mbatia, ICAP Tanzania; Geoffrey Somi, National AIDS Control Program. Al-Rahma Hospital, Bagamoyo District Hospital, Baleni Dispensary, Biharamulo Designated District Hospital, Bunazi Health Center, Bwanga Health Center, Chake Chake Hospital, Chalinze Health Center, Chato District Hospital, ChemChem (Miburani) Dispensary, Heri Mission Hospital, Ikwiriri Health Center, Isingiro Hospital, Izimbya Hospital, Kabanga Mission Hospital, Kagera Sugar Hospital, Kagondo Hospital, Kahororo Dispensary, Kaigara Health Center, Kakonko Health Center, Kanazi Health Centre, Kasulu District Hospital, Katoro Health Centre, Kayanga Health Centre, Kibiti Health Center, Kibondo District Hospital, Kigarama Health Centre, Kigoma Dispensary, Kigoma Regional Hospital, Kilimahewa Mission Dispensary, Kirongwe Dispensary, Kisarawe District Hospital, Kishanje Health Centre, Kisiju Health Centre, Kivunge Hospital, Kongowe Dispensary, Lugoba Health Center, Mabamba Health Center, Mafia District Hospital, Maneromango Health Center, Masaki Health Centre, Mchukwi Hospital, Michiweni Hospital, Miono Health Centre, Mkamba Health Centre, Mkoani Health Centre, Mkomaindo Hospital, Mkuranga District Hospital, Mlandizi Health Center, Mnazi Mmoja Hospital, Mugana Designated District Hospital, Murgwanza Designated District Hospital, Murongo Health Center, Mwembeladu Maternity Hospital, Mzenga Health Centre, Ndanda Hospital, Ndolage Hospital, Newala Hospital, Nguruka Health Centre, Nkwenda Health Center, Nyakahanga Designated District Hospital, Nyamiaga Health Centre, Ocean Road Cancer Institute, Rubya Designated District Hospital, Rulenge Hospital, Rwamishenye Health Centre, St. Therese Bukoba Health Center, Tumbi Regional Hospital, Ujiji Health Center, Utende Dispensary, Utete District Hospital, Uvinza Dispensary, Wete Hospital, Zam Zam Health Centre. *ICAP Central* - Matthew Lamb, Denis Nash, Harriet Nuwagaba-Biribonwoha
