# Supplementary material for: The epidemiology of adolescents living with perinatally acquired HIV: A cross-region global cohort analysis
Source: PLoS Med. 2018 Mar 1;15(3):e1002514. doi: 10.1371/journal.pmed.1002514 (PMC5832192; doi:10.1371/journal.pmed.1002514)
Supplement: S1 Analysis Plan — (PDF) [file pmed.1002514.s004.pdf]

CIPHER collaborative cohort project on perinatally HIV-infected adolescents

Version 0.4, 23 September 2013

CONCEPT SHEET FOR MULTI-REGIONAL COLLABORATIVE COHORT

**Title**	Global epidemiology of adolescents with perinatal HIV infection
**Proposed collaborators**	The following paediatric cohorts/collaborations are proposed as contributors of data: EPPICC, MSF, Optimal Models (ICAP), IMPAACT, PHACS, Baylor and IeDEA (East Africa, West Africa, Central Africa, Southern Africa, Asia-Pacific and CCASAnet)
**Abstract**	• *Background and objectives:* Access to combination antiretroviral therapy (cART) has led to an emerging population of perinatally HIV-infected adolescents, initially in high-income countries (Europe and USA), then progressively in Asia, South America and, most recently, sub-Saharan Africa. Although they share common features, they come from different settings. There is a global need to describe temporal trends and characteristics of these adolescents and their ability to remain in paediatric care and transition to adult care to ensure optimal health outcomes and long-term retention in care. The Collaborative Initiative for Paediatric HIV Education and Research (CIPHER) provides a unique opportunity to describe the global epidemiology of perinatally HIV-infected adolescents and compare outcomes across regional- and patient-level contexts.<br>• *Methods:* This will take the form of an international individual pooled analysis of retrospective data from all participating paediatric cohorts and cohort collaborations able to provide an electronic database. The following paediatric cohorts will be invited to contribute data: EPPICC, MSF, Optimal Models/ICAP, IMPAACT, PHACS, Baylor and IeDEA (East Africa, West Africa, Central Africa, Southern Africa, Asia-Pacific and CCASAnet). Other paediatric cohorts and cohort collaborations may also join, depending on predefined eligibility criteria. Adolescents will be defined as those entering HIV-related care by the age of 10 years (i.e., assumed perinatal infection) and continuing in follow up beyond the age of 10 years.<br>• *Anticipated results:* We aim to produce a descriptive analysis of the global epidemiology of perinatally HIV-infected adolescents by region and by age group (baseline, ART start, age 10 years) within various calendar periods, with focus on survival, retention in paediatric care and, if possible, transition to adult care.<br>• *Anticipated conclusions:* We expect to characterize survival, retention in care and transition to adult care rates according to calendar year, country, clinic, type of study (observational cohort/ clinic database vs. research cohort) and patient characteristics, stratified by age.
**Background, including any preliminary work undertaken**	Worldwide, around 330,000 infants were infected perinatally in 2011(1). These HIV-infected children now live longer because of greater access to cART and they are reaching adolescence. Perinatally HIV-infected adolescents are an emerging and growing population with specific features, observed initially in high-income countries (Europe and USA), then progressively in middle-income countries (Asia and South America) and, more recently, in low-income countries (sub-Saharan Africa) (2-10). So far, we have limited understanding of the patient- and regional-level factors associated with long-term care of these HIV-infected adolescents in these different settings. A description of their socio-medical characteristics and their ability to remain in paediatric care, as well as research on the temporal trends of these characteristics and their consequences on transitioning to adult

	care, are also needed.<br><br>Within CIPHER, a meeting of paediatric cohorts and cohort collaborations was held in Venice in May 2013, during which the need for a global analysis of perinatally HIV-infected adolescents was identified. This forum and the associated collaboration provides a unique opportunity to bring together multiple, large data sources to better describe the global epidemiology of adolescent HIV and compare programme and clinical outcomes in both regional- and individual-level contexts.
**Key aims/research questions**	**Objectives**<br>The primary objective of this study is to describe the global epidemiology, geographical and temporal trends of characteristics of perinatally HIV-infected adolescents according to baseline variables at entry into HIV care, at cART initiation, and at entry to adolescence (i.e., from the age of 10 years). These variables will capture demographic data, clinical status, antiretroviral therapy outcomes and laboratory monitoring, by region, site and study.<br><br>The secondary objective of this study is to evaluate the HIV disease status (CDC/WHO stage), and rates and predictors of death, loss to follow up (LTFU), loss to programme (death or LTFU) and transition to adult care outcomes from the age of 10 (baseline time for entry into the adolescent cohort) by regional-, site-, study- and individual-level characteristics.<br><br>**Study design:** This will take the form of an international pooled analysis of individual-level retrospective data from eligible paediatric cohort collaborations and individual cohorts, including: EPPICC, MSF, Optimal Models/ICAP, IMPAACT, PHACS, Baylor and IeDEA (East Africa, West Africa, Central Africa, Southern Africa, Asia-Pacific and CCASAnet).
**Justification for use of CIPHER collaboration**	Within CIPHER, a meeting of paediatric cohorts and cohort collaborations was held in Venice in May 2013, during which the need for a global analysis of perinatally infected adolescents was identified. This forum provides a unique opportunity to describe the global epidemiology of adolescents infected perinatally with HIV and to compare their retention in care and transition to adulthood care within both regional- and individual-level contexts.
**Study population, including estimated sample size if possible**	**Cohort eligibility criteria:**<br>Cohorts must have data available in an electronic database in 2013 in order to participate.<br><br>**Patient eligibility criteria:**<br>Participants will be HIV-infected children entering HIV care at any age before 10 years of age (proxy for perinatal infection) and followed beyond the age of 10 (follow-up data would be included up to age 19 years, encompassing the period of adolescence as defined by WHO).<br>**Exclusions:**<br>• Children with known horizontally acquired infection.

**Data required**	**Key variables and definitions:**<br>Not all variables will be available from all cohorts. Viral load data may be missing for several cohorts.<br><br>Potential overlaps would have to be identified at the regional level before data transfer to the data management center.<br><br>*Site-level variables, collected from a simple site survey at the time of data transfer as a specific site-level survey, was considered as not feasible in the timeline.*<br><br>*Patient-level data (from cohort databases) (HICDEP format):*<br><br><u>tblBAS (basic clinical, background & demographic info)</u><br>• PATIENT (cohort patient ID)<br>• CENTER (code for clinic the patient was seen at)<br>• BIRTH_D (date of birth)<br>• FRSVIS_D (first seen at clinic date)<br>• ENROL_D (date enrolled into cohort)<br>• GENDER (gender/sex)<br>• MODE (mode of transmission) (to confirm perinatal)<br>• Method of presentation<br>• ORIGIN (nationality or region of origin of patient)<br>• ETHNIC (ethnicity/race)<br>• Exposure to PMTCT<br>• RECART_Y (has patient received ART?)<br>• AIDS_Y (has patient ever had an AIDS diagnosis?)<br>• AIDS_D (if yes, date of AIDS diagnosis)<br>• WHO4_Y (has patient ever had a WHO stage 4?)<br>• WHO4_D (if yes, date of WHO stage 4?)<br>• Orphan status at entry into care<br><u>tblLTFU (death and drop-out)</u><br>• PATIENT (cohort patient ID)<br>• DROP_Y (has the patient dropped out?)<br>• DROP_D (if yes, date of last visit)<br>• DROP_RS (if yes, reason [codes here: http://www.hicdep.org/wiki/Hicdep_1.60/TableLtfu/FieldDropRs#CodingTable])<br>• DEATH_Y (has patient died?)<br>• DEATH_D (death date)<br>• DEATH_R1 (cause of death)<br>• DEATH_RC1 (causal relation of code given in DEATH_R1 to the death)<br>• DEATH_R#... (cause of death)<br>• DEATH_RC#... (causal relation of code given in DEATH_R# to the death)<br><u>tblOVERLAP(patient participation in >1 cohort)</u><br>• PATIENT (cohort patient ID)<br>• COHORT (code/ name of cohort)<br>• PAT_OTH (unique patient ID in other cohort)<br>• COH_OTH (name of other cohort)<br><u>tblVIS (basic follow-up data beyond the age of 10)</u><br>• PATIENT (cohort patient ID)<br>• VIS_D (visit date)<br>• WEIGH (weight)<br>• HEIGH (height)<br><u>tblART (ART)</u>

- PATIENT (cohort patient ID)
- ART_ID (ART drug)
- ART_SD (ART start date)
- ART_ED (ART end date)
- ART_RS (ART reason for stopping)

tblDIS (OIs) : could be an optional variable

- PATIENT (cohort patient ID)
- DIS_ID (event ID)
- DIS_D (date of event)

tblLAB_CD4

- PATIENT (cohort patient ID)
- CD4_D (CD4 date)
- CD4_V (CD4 value)
- CD4_U (CD4 unit of measurement – count/%)

tblLAB_RNA

- PATIENT (cohort patient ID)
- RNA_D (date of sample)
- RNA_V (value)
- RNA_L (lower limit of assay)
- RNA_T (type of assay)

PROPHYLAXIS

- Cotrimoxazole prophylaxis (yes/no)
- Cotrimoxazole dates starting and stopping

Outcomes: (derived from patient-level data)

- Death
- Loss to follow up (last seen >6, 9, 12, months) at the time of database closure. Sensitivity analyses will be conducted to optimize the definition.
- Loss to programme (death or loss to follow up) or retention in paediatric care
- Transfer out to other clinic
- Transfer out to adult care

Statistical methods:

- Descriptive statistics at baseline (closer visit from the age of 10) and at last follow-up
  - Age at presentation & last follow-up,
  - Year of presentation
  - Sex
  - Ethnicity
  - Weight for age z-score at presentation & last follow-up
  - Height for age z-score at presentation & last follow-up
  - CD4 count at presentation & last follow-up
  - summary WHO stage / CDC Classification
  - TB diagnosis
  - Method of presentation (prospectively from birth; VCT; symptoms etc)
  - History of PMTCT prophylaxis

- HIV care (ART and cotrimoxazole) characteristics at baseline & last follow-up,
  - % not on ART/ % previous mono or dual/ % started cART naïve (i.e. with no ART exposure prior to start of 3-drug regimen)
  - ART status & reasons for stopping
  - Drug class of initial and last regimen

	last follow up<br>- Age at presentation and last follow up<br>- Year of presentation<br>- Sex<br>- Ethnicity<br>- Weight for age z-score at presentation and last follow up<br>- Height for age z-score at presentation and last follow up<br>- CD4 count at presentation and last follow up<br>- summary WHO stage/CDC classification<br>- TB diagnosis<br>- Method of presentation (prospectively from birth, VCT, symptoms, etc.)<br>- History of PMTCT prophylaxis<br><br>• HIV care (ART and cotrimoxazole) characteristics at baseline and last follow up<br>- % not on ART / % previous mono or dual / % started cART naïve (i.e., with no ART exposure prior to start of three-drug regimen)<br>- ART status and reasons for stopping<br>- Drug class of initial and last regimen<br>- Number of drugs exposed to from cART initiation<br>- Cotrimoxazole status<br>- Median time on cART since initiation<br>- Median time on cotrimoxazole since initiation<br><br>• Status at last follow up<br>- Died<br>- LTFU<br>- Transferred to adult care or other care<br>- Alive on care<br><br>Baseline characteristics at the age of 10 (including history of HIV care) will be reported as median (IQR) for continuous variables and counts (percentage) for categorical variables.<br><br>All the above descriptions will be done globally, then stratified by calendar at first visit period (before 2004; 2004-2008; 2009-2013) and by region (geographical and according to the income status of countries). Comparisons will be carried out for some of the characteristics of interest using Chi-square, ANOVA methods or non-parametric equivalence.<br><br>• Time to event analysis using Kaplan-Meier survival analysis with delayed entry given survival to the age of 10 years (adapted for left truncation), global, and stratified by region and by time periods:<br>- To ART initiation (for those not starting before 10 years of age)<br>- To death<br>- To transfer out to adult care<br>- To loss to follow-up<br><br>• A Cox proportional hazard model analysis from the age of 10 will be conducted to analyse the predictors of the study outcomes (death/LTFU/transfer to adult care). This analysis will be done using a competing risk analysis between death and LTFU.<br><br>• Univariable analysis of predictors of death and death/LTFU (patient-level

	variables are at baseline (age of 10 years):<br>o Sex<br>o Weight for age z-score<br>o Height for age z-score<br>o CD4 count<br>o WHO stage/CDC classification<br>o TB diagnosis at entry<br>o Cotrimoxazole prophylaxis<br>o ART regimen (no ART/<6 months/≥6months)<br>o Cohort<br>o Country or income level country (high, middle, low income)<br>o Type of institution<br>o Date of ART initiation for those on ART<br><br>• Multivariate analyses will be based on the Cox proportional hazards model to summarize predictors associated with survival and retention in care and using a stepwise descendant approach, including variables that were selected by univariate analyses at the 25% level. When explanatory variables are available, the Poisson model and the Cox model with delayed entry may be used for estimating relative risks (11).<br><br>Mixed models will be used to account for clustering at multiple levels (country, site/city and, possibly, time period). Sensitivity analyses will be conducted using different LTFU definitions and ways of dealing with missing data: exclusion, inclusion as a missing category, or inclusion using missing data imputation.
**Ethical issues**	This study will use site-level data and retrospective data from individual cohorts with previous IRB approval for data collection and analysis. Only anonymized data will be transferred to the data management centre.
**Resources required (e.g., statistical support)**	Identification of a data management centre: late October 2013
**Deliverables and timelines**	- Submission to the individual cohort consortium groups (including IeDEA Pediatric Working Group): 22 June 2013<br>- Approval by the IeDEA Pediatric Working Group and other cohort consortium: 5 July 2013; conference call: 23 July 2013<br>- Approval by the CIPHER cohort collaboration and agreement on data availability survey and analysis plans between collaboration cohorts: September 2013<br>- Circulation for regional review and approval: October 2013<br>- CIPHER Executive Committee decision about data transfer centre: end of October 2013<br>- Data transfer procedures and sharing pooling from collaboration cohorts: November to March 2014<br>- Analysis completed: May 2014<br>- First draft paper June 2014 to be circulated
**References**	1. UNAIDS. UNAIDS report on the global AIDS epidemic. Geneva: UNAIDS; 2012.<br>2. Foster C, Waelbrouck A, Peltier A. Adolescents and HIV infection. Current opinion in HIV and AIDS. 2007;2(5):431-6.<br>3. Foster C, Fidler S. Optimizing antiretroviral therapy in adolescents with perinatally acquired HIV-1 infection. Expert Rev Anti-Infect Ther. 2010;8(12):1403-16.<br>4. Coovadia H, Mantell JE. Adolescents and HIV in Sub-Saharan Africa: A Timely Issue and Missed Opportunity. Clin Infect Dis. 2010;51(7):852-4.<br>5. Patel K, Hernan MA, Williams PL, Seeger JD, McIntosh K, Dyke RB, et al. Long-

	term effects of highly active antiretroviral therapy on CD4+ cell evolution among children and adolescents infected with HIV: 5 years and counting. Clin Infect Dis. 2008;46(11):1751-60.<br>6. Ryscavage PAMD, Anderson EJMD, Sutton SHMD, Reddy SP, Taiwo BM. Clinical Outcomes of Adolescents and Young Adults in Adult HIV Care. Jaids Journal of Acquired Immune Deficiency Syndromes. 2011.<br>7. Gray GE. Adolescent HIV--cause for concern in Southern Africa. PLoS Med. 2010;7(2):e1000227.<br>8. Judd A, Doerholt K, Tookey PA, Sharland M, Riordan A, Menson E, et al. Morbidity, mortality, and response to treatment by children in the United Kingdom and Ireland with perinatally acquired HIV infection during 1996-2006: planning for teenage and adult care. Clin Infect Dis. 2007;45(7):918-24. Epub 2007/09/07.<br>9. Judd A, Ferrand RA, Jungmann E, Foster C, Masters J, Rice B, et al. Vertically acquired HIV diagnosed in adolescence and early adulthood in the United Kingdom and Ireland: findings from national surveillance. HIV medicine. 2009;10(4):253-6. Epub 2009/02/04.<br>10. Van Dyke RB, Patel K, Siberry GK, Burchett SK, Spector SA, Chernoff MC, et al. Antiretroviral treatment of US children with perinatally acquired HIV infection: temporal changes in therapy between 1991 and 2009 and predictors of immunologic and virologic outcomes. J Acquir Immune Defic Syndr. 2011;57(2):165-73. Epub 2011/03/17.<br>11. Commenges D, Letenneur L, Joly P, Alioum A, Dartigues JF. Modelling age-specific risk: application to dementia. Stat Med. 1998 Sep 15;17(17):1973-88.
**Working group drafting this concept note**	Chairs: Ali Judd (EPPICC) and Valériane Leroy (IeDEA West Africa/French ANRS INSERM)<br>Members: Chloe Teasedale (Optimal Models (ICAP)), Mark Cotton (IeDEA Southern Africa), Mary-Anne Davies (IeDEA Southern Africa), Rohan Hazra (US National Institutes of Health, NICHD), George Seage (PHACS), Russell Van Dyke (IMPACT), Jihane Ben-Farhat (MSF), Jorge Pinto (CCASAnet), Colette Smith (EPPICC), Annette Sohn (IeDEA Asia-Pacific), Marcel Yotebeing (IeDEA Central Africa), Kara Wools-Kaloustian (IeDEA East Africa)
