# Supplementary material for: The epidemiology of adolescents living with perinatally acquired HIV: A cross-region global cohort analysis
Source: PLoS Med. 2018 Mar 1;15(3):e1002514. doi: 10.1371/journal.pmed.1002514 (PMC5832192; doi:10.1371/journal.pmed.1002514)
Supplement: S1 Table — (DOCX) [file pmed.1002514.s005.docx]

S1 Table: Estimates of cumulative mortality at age 15 years estimated using the Kaplan-Meier product limit estimator

|  | Cumulative mortality (%) | 95% Confidence Interval |
| --- | --- | --- |
| Total Cohort | 3.0 | 2.8; 3.3 |
|  |  |  |
| By Region |  |  |
| Europe | 0.8 | 0.5; 1.2 |
| North America | 1.1 | 0.5; 2.3 |
| South & Southeast Asia | 3.0 | 2.1; 4.3 |
| South America & Caribbean | 4.7 | 3.3; 6.6 |
| Sub-Saharan Africa | 3.5 | 3.2; 3.8 |
|  |  |  |
| By Country Income Group |  |  |
| Low Income | 4.0 | 3.6; 4.5 |
| Lower Middle Income | 3.1 | 2.4; 3.3 |
| Upper Middle Income | 1.8 | 1.4; 2.4 |
| High Income | 0.9 | 0.6; 1.4 |
|  |  |  |
| By Birth Cohort |  |  |
| Pre-1995 | 2.3 | 1.8; 3.0 |
| 1995-1999 | 3.6 | 3.3; 4.0 |
| 2000-2005 | 2.2 | 1.7; 2.7 |
