# Supplementary material for: The epidemiology of adolescents living with perinatally acquired HIV: A cross-region global cohort analysis
Source: PLoS Med. 2018 Mar 1;15(3):e1002514. doi: 10.1371/journal.pmed.1002514 (PMC5832192; doi:10.1371/journal.pmed.1002514)
Supplement: S2 Table — (DOCX) [file pmed.1002514.s006.docx]

S2 Table: Adolescent characteristics at first visit, ART start, age 10 years and last visit and cumulative incidence of outcomes (mortality, transferred out, lost-to-follow-up) compared by birth cohort

|  | | Total | | Pre-1995 | | 1995-1999 | | 2000-2005 |
| --- | --- | --- | --- | --- | --- | --- | --- | --- |
| Total N (row %) | | 38187  (100) | | 2660  (7.0) | | 13267  (34.7) | | 22260  (58.3) |
| Region – N (%) | |  | |  | |  | |  |
| Europe | | 3054 (8.0) | | 1399 (52.6) | | 989 (7.5) | | 666 (3.0) |
| North America | | 1032 (2.7) | | 640 (24.1) | | 318 (2.4) | | 74 (0.3) |
| South & Southeast Asia | | 2902 (7.6) | | 91 (3.4) | | 918 (6.9) | | 1893 (8.5) |
| South America & Caribbean | | 903 (2.4) | | 182 (6.8) | | 446 (3.4) | | 275 (1.2) |
| Sub-Saharan Africa | | 30296 (79.3) | | 348 (13.1) | | 10596 (79.9) | | 19352 (86.9) |
| Country Income Group N (%) | |  | |  | |  | |  |
| Low | | 24794 (64.9) | | 229 (8.6) | | 8172 (61.6) | | 16393 (73.6) |
| Lower Middle | | 3015 (7.9) | | 326 (12.3) | | 1087 (8.2) | | 1602 (7.2) |
| Upper Middle | | 6669 (17.5) | | 289 (10.9) | | 2729 (20.6) | | 3651 (16.4) |
| High | | 3709 (9.7) | | 1816 (68.3) | | 1279 (9.6) | | 614 (2.8) |
| Male – N (%) | | 18863 (49.4) | | 1305 (49.1) | | 6472 (48.8) | | 11086 (49.8) |
| Age in years – median (IQR) | |  | |  | |  | |  |
| First visit | | 6.7 (4.4; 8.4) | | 2.7 (0.4; 7.2) | | 7.8 (6.1; 9.0) | | 6.1 (4.2; 8.0) |
| ART start | | 7.5 (5.2; 9.2) | | 5.3 (1.6 ; 9.3) | | 8.4 (6.7; 9.6) | | 7.0 (4.8; 8.7) |
| Last visit | | 12.4 (11.1; 14.4) | | 17.6 (15.0; 18.7) | | 14.6 (12.9; 15.9) | | 11.5 (10.7; 12.6) |
| CD4 count in cells/mm^3^ – median (IQR) | |  | |  | |  | |  |
| First visit all ages [N=19979] | | 427 (200; 757) | | 768 (322; 1520) | | 359 (153; 650) | | 454 (227; 778) |
| First visit if age > 5 years [N=14585] | | 358 (165; 632) | | 268 (103; 515) | | 317 (132; 560) | | 391 (197; 683) |
| ART start [N=20608] | | 321 (165; 575) | | 450 (190; 957) | | 285 (135; 494) | | 336 (184; 590) |
| ART start if age > 5 years [N=16612] | | 292 (161; 469) | | 258 (116; 466) | | 264 (122; 431) | | 314 (171; 530) |
| Age 10 years [N=26953] | | 685 (445; 972) | | 610 (365; 898) | | 625 (395; 899) | | 736 (489; 1024) |
| Last visit [N=31951] | | 687 (464; 946) | | 569 (378; 800) | | 631 (419; 871) | | 742 (507; 1011) |
| CD4 % - median (IQR) | |  | |  | |  | |  |
| First visit [N=13674] | | 16 (9; 25) | | 26 (15; 36) | | 15 (8; 23) | | 16 (10; 24) |
| ART start [N=14740] | | 14 (8 ; 20) | | 19 (11; 29) | | 13 (7; 19) | | 14 (8.3; 19) |
| Age 10 years [N=17974] | | 28 (20; 34) | | 26 (18; 34) | | 26 (19; 32) | | 29 (22; 36) |
| Last visit [N=23292] | | 29 (21; 35) | | 28 (19; 34) | | 28 (20; 34) | | 29 (22; 36) |
| HAZ – median (IQR) | |  | |  | |  | |  |
| First visit[N=20269] | | -1.92 (-2.91; -0.97) | | -1.14 (-2.21; -0.17) | | -1.88 (-2.81; -0.96) | | -1.99 (-3.01; -1.04) |
| ART start [N=20372] | | -1.95 (-2.91; -1.02) | | -1.22 (-2.15; -0.31) | | -1.93 (-2.83; -1.03) | | -2.03 (-3.02; -1.11) |
| Age 10 years [N=26883] | | -1.53 (-2.35; -0.72) | | -0.70 (-1.60; 0.11) | | -1.63 (-2.46; -0.75) | | -1.56 (-2.35; -0.81) |
| Last visit [N=32752] | | -1.59 (-2.45; -0.72) | | -0.65 (-1.44; 0.09) | | -1.59 (-2.47; -0.72) | | -1.7 (-2.51; -0.86) |
| ART – N (%) | |  | |  | |  | |  |
| Ever received | | 33514 (87.8) | | 2512 (94.4) | | 11883 (89.6) | | 19119 (85.9) |
| Started age > 10 years | | 4037 (12.0) | | 420 (16.7) | | 2029 (17.1) | | 1588 (8.3) |
| On ART at age 10 years | | 25713 (67.3) | | 1640 (61.7) | | 8562 (64.5) | | 15511 (69.7) |
| On ART at last visit | | 30072 (80.3) | | 2065 (80.4) | | 10589 (82.5) | | 17418 (78.9) |
| Virologic suppression – n/N (%) | | | | | | | | |
| Age 10 years | 6919/10209 ( 67.8) | | 794/1882 (42.2) | | 2803/3938 (71.2) | | 3322/4389 (75.7) | |
| Last visit | 9741/14200 (68.6) | | 1396/2378 (58.7) | | 3803/5513 (69.0) | | 4542/6309 (72.0) | |
| Cumulative incidence (95% CI) at age 15 years | | | | | | | | |
| Mortality (%) | 2.6 (2.4; 2.8) | | 2.3 (1.77; 2.94) | | 3.19 (2.88; 3.51) | | 1.84 (1.50; 2.23) | |
| Transferred out (%) | 15.6 (15.1; 16.0) | | 3.63 (2.94; 4.42) | | 14.96 (14.32; 15.61) | | 22.53 (20.64; 24.47) | |
| Lost to follow-up (%) | 11.3 (10.9; 11.8) | | 4.87 (4.07; 5.77) | | 10.48 (9.92; 11.05) | | 23.34 (19.96; 26.88) | |

ART – antiretroviral therapy; CI – confidence interval; HAZ – WHO height-for-age Z-score; IQR – interquartile range
