# Supplementary material for: The epidemiology of adolescents living with perinatally acquired HIV: A cross-region global cohort analysis
Source: PLoS Med. 2018 Mar 1;15(3):e1002514. doi: 10.1371/journal.pmed.1002514 (PMC5832192; doi:10.1371/journal.pmed.1002514)
Supplement: S3 Table — (DOCX) [file pmed.1002514.s007.docx]

S3 Table: Cumulative incidence (95%CI) for outcomes between 10 and 13 years of age by birth cohort stratified by region and by country income group

|  | Total | Pre-1995 | 1995-1999 | 2000-2005 |
| --- | --- | --- | --- | --- |
| Stratified by Region | | | | |
| Europe | N=3054 | N=1399 | N=989 | N=666 |
| Mortality (%) | 0.47 (0.26; 0.78) | 0.72 (0.37; 1.28) | 0.30 (0.09; 0.85) | 0 |
| Transfer Out (%) | 1.33 (0.95; 1.81) | 0.64 (0.32; 1.19) | 1.52 (0.89; 2.45) | 3.87 (2.01; 6.68) |
| LTFU (%) | 2.79 (2.22; 3.46) | 1.07 (0.63; 1.73) | 1.73 (1.05; 2.70) | 12.73 (9.29; 16.74) |
| North America | N=1032 | N=640 | N=318 | N=74 |
| Mortality (%) | 0.55 (0.21; 1.22) | 0.82 (0.31; 1.82) | 0 | 0 |
| Transfer Out (%) | 1.28 (0.68; 2.22) | 0.88 (0.34; 1.95) | 1.69 (0.55; 4.08) | 3.23 (0.60; 9.99) |
| LTFU (%) | 4.27 (3.07; 5.85) | 3.72 (2.38; 5.52) | 5.68 (2.89; 9.80) | 6.14 (1.56; 15.38) |
| South & Southeast Asia | N=2902 | N=91 | N=918 | N=1893 |
| Mortality (%) | 1.24 (0.82; 1.80) | 5.50 (2.04; 11.50) | 1.20 (0.64; 2.08) | 0.87 (0.43; 1.63) |
| Transfer Out (%) | 4.65 (3.80; 5.64) | 0 | 6.11 (4.68; 7.78) | 3.62 (2.57; 4.93) |
| LTFU (%) | 2.73 (2.04; 3.57) | 2.20 (0.42; 6.95) | 1.86 (1.13; 2.89) | 4.10 (2.75; 5.85) |
| South America & Caribbean | N=903 | N=182 | N=446 | N=275 |
| Mortality (%) | 2.19 (1.35; 3.38) | 2.75 (1.03; 5.92) | 2.91 (1.63; 4.79) | 0 |
| Transfer Out (%) | 4.47 (3.18; 6.09) | 3.85 (1.70; 7.38) | 5.38 (3.54; 7.75) | 2.80 (0.86; 6.83) |
| LTFU (%) | 1.89 (1.11; 3.03) | 1.65 (0.45; 4.40) | 2.08 (1.00; 3.66) | 1.63 (0.44; 4.37) |
| Sub-Saharan Africa | N=30296 | N=348 | N=10596 | N=19352 |
| Mortality (%) | 1.92 (1.74; 2.11) | 3.16 (1.68;5.40) | 2.42 (2.14; 2.73) | 1.49 (1.26; 1.74) |
| Transfer Out (%) | 12.83 (12.39; 13.29) | 7.19 (4.79; 10.22) | 11.17 (10.57; 11.79) | 14.65 (13.95; 15.38) |
| LTFU (%) | 7.25 (6.90; 7.63) | 5.18 (3.19; 7.86) | 6.65 (6.18; 7.15) | 8.29 (7.70; 8.92) |
|  |  |  |  |  |
| Stratified by Country Income Group | | | | |
| High Income | N=3,709 | N=1,816 | N=1,279 | N=614 |
| Mortality (%) | 0.50 (0.33; 0.83) | 0.85 (0.50; 1.37) | 0.25 (0.07; 0.70) | 0 |
| Transfer Out (%) | 1.35 (1.00; 1.79) | 0.80 (0.46; 1.31) | 1.60 (1.00; 2.44) | 3.58 (1.57; 5.47) |
| LTFU (%) | 3.07 (2.52; 3.71) | 1.96 (1.38; 2.69) | 2.38 (1.61; 3.40) | 11.15 (7.95; 14.93) |
| Upper Middle Income | N=6669 | N=289 | N=2729 | N=3651 |
| Mortality (%) | 0.78 (0.57; 1.05) | 1.73 (0.66; 3.78) | 0.90 (0.49; 1.32) | 0.51 (0.28; 0.88) |
| Transfer Out (%) | 15.48 (14.51; 16.48) | 5.19 (3.04; 8.17) | 13.95 (12.66; 15.30) | 17.92 (16.33; 19.56) |
| LTFU (%) | 6.17 (5.52; 6.88) | 2.42 (1.08; 4.71) | 4.13 (3.41; 4.94) | 9.89 (8.53; 11.35) |
| Lower Middle Income* | N=3015 | N=326 | N=1087 | N=1602 |
| Mortality (%) | 1.77 (1.30; 2.36) | 1.53 (0.58; 3.36) | 2.21 (1.46; 3.22) | 1.56 (0.86; 2.62) |
| Transfer Out (%) | 10.49 (9.30; 11.75) | 0 | 10.43 (8.70; 12.34) | 13.70 (11.58; 16.01) |
| LTFU (%) | 3.67 (2.95; 4.50) | 0.92 (0.26; 2.50) | 3.23 (2.30; 4.41) | 5.49 (4.03; 7.27) |
| Low Income | N=24794 | N=229 | N=8172 | N=16393 |
| Mortality (%) | 2.18 (1.96; 2.40) | 4.81 (2.55; 8.14) | 2.83 (2.48; 3.20) | 1.61 (1.35; 1.91) |
| Transfer Out (%) | 10.79 (10.32; 11.26) | 7.43 (4.51; 11.30) | 9.40 (8.77; 10.05) | 12.38 (11.65; 13.14) |
| LTFU (%) | 7.21 (6.80; 7.63) | 6.56 (3.84; 10.27) | 7.12 (6.57; 7.70) | 7.57(6.92; 8.25) |

CI – confidence interval; LTFU – lost to follow-up; * Lower middle income group had a marked difference in distribution by country across the birth cohorts: pre-1995 68% from Romania, 27% Thailand; 1995-1999 21% from Lesotho, 16% Swaziland, 48% Thailand; 2000-2005 35% from Lesotho, 24% Swaziland, 24% Thailand
