# Supplementary material for: The epidemiology of adolescents living with perinatally acquired HIV: A cross-region global cohort analysis
Source: PLoS Med. 2018 Mar 1;15(3):e1002514. doi: 10.1371/journal.pmed.1002514 (PMC5832192; doi:10.1371/journal.pmed.1002514)
Supplement: S4 Table — (DOCX) [file pmed.1002514.s008.docx]

S4 Table: Selected complete multivariable models: mortality hazard ratios and 95% confidence intervals

|  | uHR | uHR with MI (N=38,187) | aHR  without IPW or MI  (N=38,187) | aHR  with IPW  (N=38,187) | aHR  without MI  (N=13,699) | aHR  with MI  (N=38,187) |
| --- | --- | --- | --- | --- | --- | --- |
| Region (N=38,187) |  |  |  |  |  |  |
| Europe | 1.00 | 1.00 | 1.00 | 1.00 | 1.00 | 1.00 |
| North America | 1.70 (0.87; 3.31) | 1.70 (0.87; 3.31) | 2.03 (1.02; 4.02) | 2.52 (1.24; 5.11) | 1.12 (0.31; 4.03) | 2.31 (1.16; 4.60) |
| South & Southeast Asia | 3.21 (2.03; 5.07) | 3.21 (2.03; 5.07) | 2.96 (1.79; 4.88) | 2.78 (1.75; 4.42) | 3.07 (1.11; 8.41) | 2.21 (1.33; 3.67) |
| South America & Caribbean | 6.07 (3.87; 9.50) | 6.07 (3.87; 9.50) | 5.94 (3.77; 9.38) | 5.61 (3.58; 8.77) | 1.69 (0.60; 4.77) | 5.21 (3.29; 8.23) |
| Sub-Saharan Africa | 4.35 (3.02; 6.28) | 4.35 (3.02; 6.28) | 3.37 (2.17; 5.23) | 3.23 (2.21; 4.73) | 4.57 (1.78; 11.71) | 2.85 (1.83; 4.43) |
| Male - reference female (N=38,187) | 1.07 (0.92; 1.24) | 1.07 (0.92; 1.24) | 1.09 (0.94; 1.26) | 1.09 (0.94; 1.24) | 0.96 (0.75; 1.24) | 1.05 (0.90; 1.22) |
| Birth cohort (N=38,187) |  |  |  |  |  |  |
| Pre-1995 | 1.00 | 1.00 | 1.00 | 1.00 | 1.00 | 1.00 |
| 1995-1999 | 1.50 (1.18; 1.91) | 1.50 (1.18; 1.91) | 0.73 (0.55; 0.98) | 0.94 (0.71; 1.24) | 0.60 (0.34; 1.05) | 0.71 (0.52; 0.95) |
| 2000-2005 | 0.94 (0.71; 1.24) | 0.94 (0.71; 1.24) | 0.51 (0.36; 0.71) | 0.69 (0.49; 0.97) | 0.44 (0.23; 0.82) | 0.48 (0.35; 0.68) |
| Age at first visit per year (N=38,187) | 1.19 (1.15; 1.23) | 1.19 (1.15; 1.23) | 1.14 (1.10; 1.19) | 1.52 (1.46; 1.59) | 1.10 (1.01; 1.20) | 1.08 (1.04; 1.31) |
| ART (N=38,187) |  |  |  |  |  |  |
| Never on ART | 1.00 | 1.00 | 1.00 | 1.00 | 1.00 | 1.00 |
| Started with dual therapy | 0.31 (0.21; 0.45) | 0.31 (0.21; 0.45) | 0.73 (0.45; 1.18) | 0.78 (0.47; 1.31) | 1.80 (0.60; 5.42) | 0.59 (0.36; 0.97) |
| Started with triple therapy | 0.57 (0.46; 0.71) | 0.57 (0.46; 0.71) | 0.62 (0.50; 0.76) | 0.55 (0.45; 0.69) | 0.35 (0.24; 0.52) | 0.45 (0.36; 0.57) |
| First visit CD4 count/50 cell increase (N=19,979) | 0.94 (0.92; 0.95) | 0.95 (0.94; 0.97) | ---------------- | ---------------- | 0.94 (0.92; 0.96) | 0.96 (0.95; 0.98) |
| Time-updated CD4 count/50 cell increase (N=32,565) | 0.82 (0.81; 0.84) | 0.87 (0.85; 0.88) | ---------------- | ---------------- | ---------------- | ---------------- |
| CD4 % at first visit (N=13,674) | 0.95 (0.94; 0.96) | 0.95 (0.94; 0.96) | ---------------- | ---------------- | ---------------- | ---------------- |
| HAZ at first visit (N=20,269) | 0.90 (0.84; 0.96) | 0.91 (0.85; 0.97) | ---------------- | ---------------- | 0.90 (0.82; 0.98) | 0.93 (0.87; 1.00) |

ART – antiretroviral therapy; HAZ – WHO height-for-age Z-score; aHR – adjusted hazard ratio; uHR – unadjusted hazard ratio; IPW – inverse probability weighting; MI – multiple imputation
