# Supplementary material for: The epidemiology of adolescents living with perinatally acquired HIV: A cross-region global cohort analysis
Source: PLoS Med. 2018 Mar 1;15(3):e1002514. doi: 10.1371/journal.pmed.1002514 (PMC5832192; doi:10.1371/journal.pmed.1002514)
Supplement: S5 Table — (DOCX) [file pmed.1002514.s009.docx]

S5 Table: Cumulative incidence (95% CI) of outcomes between 10 and 15 years of age under varying assumptions of proportion of lost to follow-up as mortality (N=38,187)

| Outcome | Europe | North America | South & Southeast Asia | South America & Caribbean | Sub-Saharan Africa |
| --- | --- | --- | --- | --- | --- |
| 1. Original estimates | | | | | |
| Mortality (%) | 0.8 (0.5; 1.2) | 1.1 (0.5; 2.1) | 2.7 (1.9; 3.8) | 4.4 (3.1; 6.1) | 2.9 (2.7; 3.2) |
| Transfer Out (%) | 3.5 (2.9; 4.3) | 1.9 (1.1; 3.1) | 6.7 (5.5; 8.0) | 6.5 (4.9; 8.5) | 19.3 (18.7; 20.0) |
| Lost to follow-up (%) | 6.1 (5.2; 7.0) | 8.9 (6.7; 11.3) | 7.1 (5.6; 8.7) | 4.8 (3.4; 6.7) | 13.2 (12.6; 13.7) |
| 1. 100% of LTFU in all regions assumed to be mortality | | | | | |
| Mortality (%) | 8.3 (7.3; 9.3) | 10.9 (8.6; 13.6) | 9.8 (8.2; 11.6) | 10.7 (8.6; 13.2) | 14.9 (14.3; 15.5) |
| Transfer out (%) | 3.6 (2.9; 4.3) | 1.9 (1.1; 3.1) | 6.7 (5.5; 8.1) | 6.6 (4.9; 8.5) | 19.7 (19.0; 20.3) |
| 1. 50% of LTFU in all regions assumed to be mortality | | | | | |
| Mortality (%) | 4.7 (3.9; 5.6) | 6.4 (4.6; 8.4) | 6.1 (4.8; 7.6) | 7.6 (5.8; 9.8) | 8.9 (8.4; 9.3) |
| Transfer Out (%) | 3.6 (2.9; 4.3) | 1.9 (1.1; 3.1) | 6.7 (5.5; 8.0) | 6.6 (4.9; 8.5) | 19.5 (18.9; 20.1) |
| Lost to follow-up (%) | 3.0 (2.4; 3.7) | 3.8 (2.5; 5.6) | 4.1 (3.1; 5.3) | 2.3 (1.4; 3.7) | 6.5 (6.1; 6.9) |
| 1. 20% of LTFU in all regions assumed to be mortality | | | | | |
| Mortality (%) | 2.5 (2.0; 3.2) | 3.1 (2.0; 4.7) | 3.9 (2.9; 5.2) | 5.6 (4.1; 7.4) | 5.3 (5.0; 5.7) |
| Transfer Out (%) | 3.6 (2.9; 4.3) | 1.9 (1.1; 3.1) | 6.7 (5.5; 8.0) | 6.6 (4.9; 8.5) | 19.4 (18.8; 20.1) |
| Lost to follow-up (%) | 4.7 (4.0; 5.6) | 6.5 (4.8; 8.6) | 5.8 (4.6; 7.3) | 3.8 (2.5; 5.5) | 10.5 (9.9; 11.0) |
| 1. 50% of LTFU in sub-Saharan Africa and 5% of LTFU in all other regions assumed to be mortality | | | | | |
| Mortality (%) | 1.3 (0.9; 1.8) | 1.4 (0.7; 2.5) | 3.0 (2.1; 4.2) | 4.7 (3.3; 6.4) | 8.9 (8.4; 9.3) |
| Transfer Out (%) | 3.6 (2.9; 4.3) | 1.9 (1.1; 3.1) | 6.7 (5.5; 8.0) | 6.5 (4.9; 8.5) | 19.5 (18.9; 20.1) |
| Lost to follow-up (%) | 5.6 (4.8; 6.6) | 8.5 (6.4; 10.9) | 6.8 (5.5; 8.4) | 4.6 (3.2; 6.4) | 6.5 (6.1; 6.9) |
| 1. 20% of LTFU in sub-Saharan Africa and 5% of LTFU in all other regions assumed to be mortality | | | | | |
| Mortality (%) | 1.3 (0.9; 1.8) | 1.4 (0.7; 2.5) | 3.0 (2.1; 4.2) | 4.7 (3.3; 6.4) | 5.3 (5.0; 5.7) |
| Transfer Out (%) | 3.6 (2.9; 4.3) | 1.9 (1.1; 3.1) | 6.7 (5.5; 8.0) | 6.5 (4.9; 8.5) | 19.4 (18.8; 20.1) |
| Lost to follow-up (%) | 5.6 (4.8; 6.6) | 8.5 (6.4; 10.9) | 6.8 (5.5; 8.4) | 4.6 (3.2; 6.4) | 10.5 (9.9; 11.0) |

CI – confidence interval; LTFU – lost to follow-up
