# Supplementary material for: The epidemiology of adolescents living with perinatally acquired HIV: A cross-region global cohort analysis
Source: PLoS Med. 2018 Mar 1;15(3):e1002514. doi: 10.1371/journal.pmed.1002514 (PMC5832192; doi:10.1371/journal.pmed.1002514)
Supplement: S6 Table — (DOCX) [file pmed.1002514.s010.docx]

S6 Table: Crude mortality hazard ratios (95% CI) by geographic region with Europe as reference, under varying assumptions of proportion of LTFU as mortality (N=38,187)

| North America | South & Southeast Asia | South America & Caribbean | Sub-Saharan Africa |
| --- | --- | --- | --- |
| 1. Original crude mortality hazard ratio | | | |
| 1.70 (0.87; 3.31) | 3.21 (2.03; 5.07) | 6.07 (3.88; 9.50) | 4.35 (3.02; 6.28) |
| 1. 100% of LTFU in all regions assumed to be mortality | | | |
| 1.29 (1.07; 1.55) | 0.84 (0.71; 1.00) | 1.07 (0.89; 1.28) | 1.46 (1.33; 1.61) |
| 1. 50% of LTFU in all regions assumed to be mortality | | | |
| 1.53 (1.21; 1.93) | 0.88 (0.70; 1.10) | 1.38 (1.10; 1.74) | 1.66 (1.45; 1.89) |
| 1. 20% of LTFU in all regions assumed to be mortality | | | |
| 1.30 (0.91; 1.84) | 1.08 (0.80; 1.45) | 1.88 (1.41; 2.51) | 1.91 (1.59; 2.29) |
| 1. 50% of LTFU in sub-Saharan Africa and 5% of LTFU in all other regions assumed to be mortality | | | |
| 1.35 (0.80; 2.29) | 1.87 (1.28; 2.72) | 3.50 (2.41; 5.07) | 7.09 (5.45; 9.23) |
| 1. 20% of LTFU in sub-Saharan Africa and 5% of LTFU in all other regions assumed to be mortality | | | |
| 1.37 (0.80; 2.33) | 1.92 (1.31; 2.79) | 3.52 (2.43; 5.11) | 4.36 (3.34; 5.70) |

CI – confidence interval; LTFU – lost to follow-up
