# Supplementary figures and images for: The epidemiology of adolescents living with perinatally acquired HIV: A cross-region global cohort analysis
Source: PLoS Med. 2018 Mar 1;15(3):e1002514. doi: 10.1371/journal.pmed.1002514 (PMC5832192; doi:10.1371/journal.pmed.1002514)

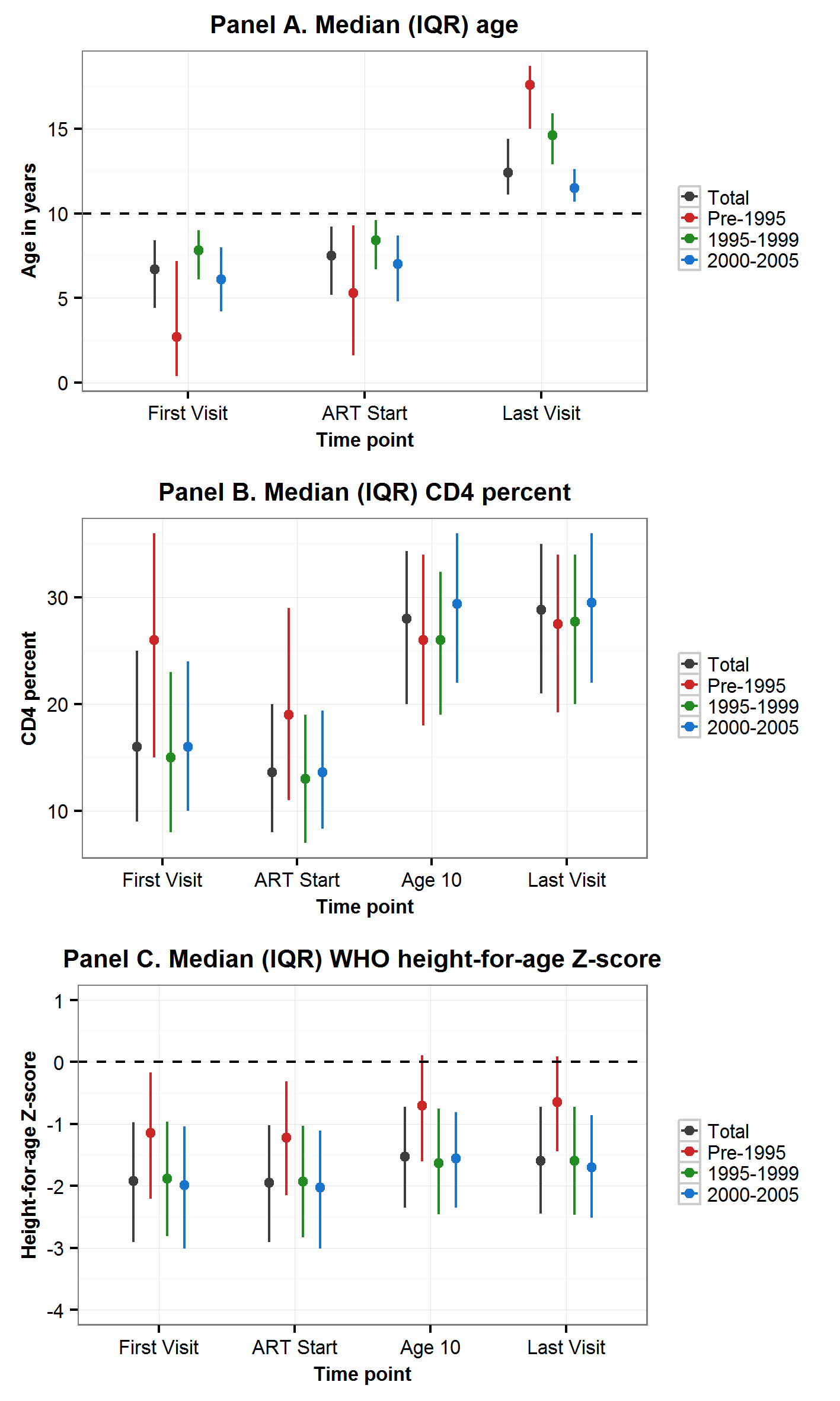

Supplement: S1 Fig — (TIFF) [file pmed.1002514.s011.tiff]
